# Historical Data Mining Deep Dive into Machine Learning-Aided 2D Materials Research in Electrochemical Applications

**DOI:** 10.1021/acsmaterialsau.5c00030

**Published:** 2025-06-23

**Authors:** Krittapong Deshsorn, Panwad Chavalekvirat, Somrudee Deepaisarn, Ho-Chiao Chuang, Pawin Iamprasertkun

**Affiliations:** † School of Bio-Chemical Engineering and Technology, Sirindhorn International Institute of Technology, 37698 Thammasat University, Pathum Thani 12120, Thailand; ‡ Research Unit in Sustainable Electrochemical Intelligent, Thammasat University, Pathum Thani 12120, Thailand; § School of Information, Computer, and Communication Technology, Sirindhorn International Institute of Technology, Thammasat University, Pathum Thani 12120, Thailand; ∥ Department of Mechanical Engineering, National Taipei University of Technology, Taipei 10608, Taiwan

**Keywords:** machine learning, 2D materials, data science, data mining, electrochemistry, KDD, energy, storage, conversion

## Abstract

Machine learning transforms the landscape of 2D materials design, particularly in accelerating discovery, optimization, and screening processes. This review has delved into the historical and ongoing integration of machine learning in 2D materials for electrochemical energy applications, using the Knowledge Discovery in Databases (KDD) approach to guide the research through data mining from the Scopus database using analysis of citations, keywords, and trends. The topics will first focus on a “macro” scope, where hundreds of literature reports are computer analyzed for key insights, such as year analysis, publication origin, and word co-occurrence using heat maps and network graphs. Afterward, the focus will be narrowed down into a more specific “micro” scope obtained from the “macro” overview, which is intended to dive deep into machine learning usage. From the gathered insights, this work highlights how machine learning, density functional theory (DFT), and traditional experimentation are jointly advancing the field of materials science. Overall, the resulting review offers a comprehensive analysis, touching on essential applications such as batteries, fuel cells, supercapacitors, and synthesis processes while showcasing machine learning techniques that enhance the identification of critical material properties.

## Graphical abstract

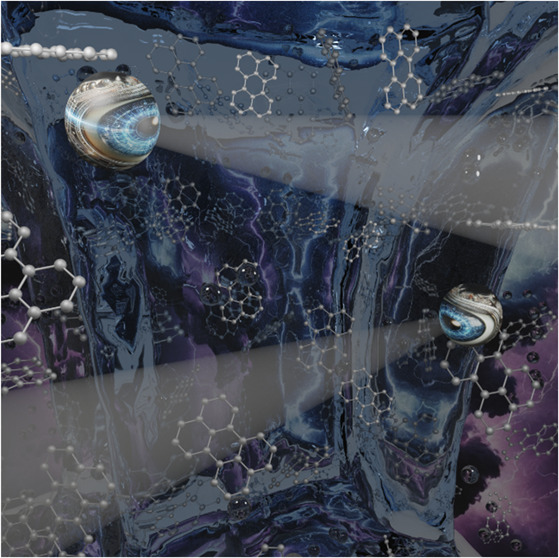

## Introduction

Research on 2D materials has advanced rapidly in recent years, with breakthroughs in materials like graphene,^1^ MXenes,^2^ and transition metal dichalcogenides (TMDs)^3^ revolutionizing various technological fields. These materials offer unique properties that are driving innovations at a remarkable pace, particularly in electrochemical applications, which are crucial for a sustainable, energy-efficient future. These unique properties of graphene and their usage in electrochemical devices for the future are especially promising due to its high surface area (>2630 m^2^ g^–1^),^4^ electrical conductivity,^5^ and customizable structure,^6^ additionally, it has “tunability”,^7^ allowing for customization for various applications (e.g., affecting conductivity,^8^ band gap^9^). MXenes, with their high electrical conductivity and mechanical strength of transition metal carbides/nitrides, combined with functionalized surfaces that render MXenes hydrophilic and capable of bonding with diverse species, their high negative zeta potential for stable colloidal solutions in water, and their effective electromagnetic wave absorption, have driven their widespread application across various fields.^2^ TMDs exhibit a range of remarkable properties, including a direct band gap, pronounced spin–orbit coupling, and outstanding electronic and optical characteristics driven by quantum confinement effects.^10^ These remarkable properties have inspired researchers to continue the discovery of new and exciting breakthroughs in this field. However, the vast and growing body of published research has made it increasingly challenging for both new and experienced researchers to stay on-topic with the latest developments. Adding to this challenge, machine learning (ML) and artificial intelligence (AI) have experienced significant growth over the past years, finding diverse applications (e.g., discovery, optimization, explanation) in 2D materials research.^11^ As a data-centric approach, ML aids in the interpretation of complex data sets by optimizing parameters (e.g., determining ideal properties for specific applications), analyzing the impact of various parameters, and revealing interactions between parameters.^12^ When combined with traditional experimental methods, ML can significantly enhance the ability to make predictions and derive insights from data^13–15^ while simultaneously improving research time and cost of research (e.g., reducing the workload of researchers from screening large and complex data sets and rapid, nonexperimental optimization of properties). However, as ML is a relatively new field incorporated into 2D materials research, some key questions arise: How is ML being applied in 2D materials research? Which materials are the focus of ML-driven studies? What trends are emerging in this rapidly evolving field? To answer these questions, a researcher must analyze hundreds of publications encompassing the entire domain, which is overwhelming. For example, following the articles from the earliest year to the current year can lead to thousands of articles that need to be assessed. Normally, a select few of the top articles with high impact are analyzed to reduce the workload and time for literature review. However, every article published in the respective field contributes to its foundation, which when neglected leads to an incomplete overview of the field (information loss). Luckily, the abstracts, keywords, and other information are readily available for bulk download from publishers (e.g., as a CSV file), which can be used to discover hidden knowledge in data. Specifically, the accumulation of these CSV files combined with Knowledge Discovery in Databases (KDD), a standardized and frequently used way of analysis and modeling of large bodies of complex data^16^ proposed by Fayyad et al. in 1996,^17^ can be followed to ensure a guided, structured method of turning large quantities of data into knowledge. Generally, the KDD method follows nine steps: (1) developing an understanding of the application domain (2) curating the appropriate data set (3) preprocessing and cleaning (4) data transformation (5) choosing the appropriate data mining task (6) choosing the data mining algorithm (7) employing the data mining algorithm (8) evaluation and (9) utilization of discovered knowledge. Following these steps ensures a structured way of knowledge discovery, which is useful when conveying findings to the general public or doing scientific communication. In addition, this flexible method of knowledge extraction can be tailored to the user’s needs while keeping an organized structure that is easily understood by all audiences.^16^ For example, Helma utilized knowledge discovery and data mining in the field of toxicology. They showcase how to perform feature calculation and selection (data set curation and data preprocessing, cleaning, and transformation), model induction and validation (choosing data mining task/algorithm), and last interpretation (evaluation and utilization) in their study.^18^ Their study demonstrates the flexibleness of the knowledge discovery process, which can be linked to the Fayyad et al. KDD method. Another applied example is by Gertosio & Dussauchoy, who utilized the KDD method to solve an industrial task, which is the reduction of the processing time in fuel production.^19^ Here, they added 2 additional steps to the KDD method (economic evaluation and industrial validation), which is a special addition for this specific task, to successfully implement a model that predicts a 28% reduction in processing time. Continuing on, this study follows the KDD workflow with some modified steps to produce a “macro” evaluation of the topic alongside a “micro” deep dive into specific, high-impact articles. This highlights both the important insights that may be missed and the high-impact publications in this domain.

Therefore, the goal of this study is to utilize KDD and data mining to provide both a macrolevel and in-depth analysis of key trends in 2D materials research, with a particular focus on the use of machine learning in electrochemical applications. By utilizing the KDD methods to analyze text data, such as publication abstracts, years, and the countries of corresponding authors from Scopus (a publication database), this work seeks to uncover the impact of ML on the direction and focus of 2D materials research. For this review, it is expected to aid in the summarization of data for new and experienced researchers alike to understand the process, usage, and application of machine learning in 2D materials research in electrochemical applications. Expectantly, readers of this review are inspired to perform research via the research of historical data to accelerate discovery and optimization, reveal new mechanisms, and even aid in the synthesis and manufacturing process.

## Methodology and Procedure of Text Mining

As mentioned, KDD can be tailored to meet the needs of the user. In this study, KDD is utilized in the steps, as seen in Figure 1. More detailed explanations can be found in their respective sections below. First, the domain is explored via the utilization of Scopus, domain knowledge, and other sources. After which, a data set is curated from exploration. In this case, the data of publications relating to the topic are downloaded from Scopus. The data are then preprocessed and cleaned to remove any stopwords, special characters, and so on. Additionally, the data are transformed (e.g., into strings, word counts). After which, the data are passed into data mining algorithms. In this study, word clouds, co-occurrence networks, and heat maps were chosen to represent and display the data. Finally, all results were evaluated and turned into a manuscript.

**Figure 1:**
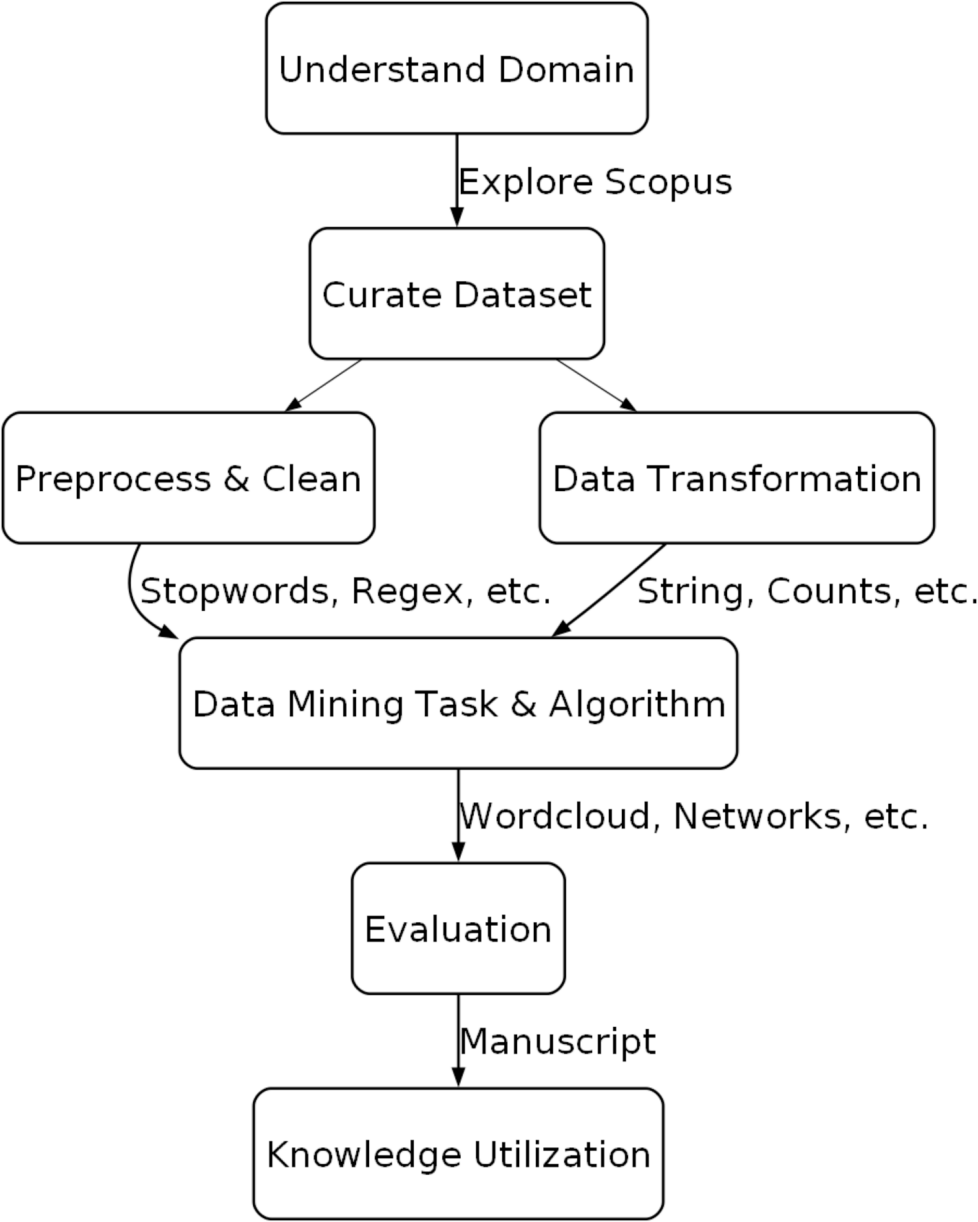
Workflow of this study, which follows the KDD process, from understanding the domain, curating the data set, preprocessing, passing through data mining algorithms, and finally utilization of the knowledge.

### Developing an Understanding of the Application Domain

The initial step of the KDD process is to define the goals and understand the application domain, which sets a foundation for further steps of the KDD process, such as preprocessing, algorithm selection, and data presentation. In this study, the scope is narrowed down to the use of machine learning (ML) in the field of 2D materials research for electrochemical energy applications. The objective is to contribute further insight into the domain for new and experienced researchers, alike, using large quantities of text data from various publications. Key elements in text data include abstracts, keywords, publication year, and corresponding author addresses, which can reveal trends over time, highlight frequently used materials, and indicate where most publications originate.

### Curating the Appropriate Data Set

After defining the goals, the data that will be used for discovery must be determined. Here, identification of where the data are located and how they are obtained is of concern. The chosen data repository is the “Scopus database”, which is a large multidisciplinary database with information about peer-reviewed publications, journals, and abstracts. Scopus provides a search query for specific keywords found in publications. The main query keywords used in this study are 2d OR ″two dimen*″ OR ″2 dimen*″ AND material* AND “machine learn*” AND elec*. The first three parts of the search query find words related to 2d or two dimen* or 2 dimen*. The asterisk is a wildcard, which matches any number of characters. This allows users to find words related to the word “dimen” (e.g., “dimension”, “dimensions”, “dimensional”). The quotation marks indicate a full word while ignoring hyphens, which will not be split during the search. For example, searching material science will yield material AND science, which finds both words, while “material science” finds the words material science adjacent to each other. The next parts search for any word related to material* and “machine learn*”. Finally, elec* searches for any terms related to electronics, electricity, electrochemistry, etc. All results are limited to articles. Remarks, the search query results *may not be all related to electrochemistry* due to a broad search yielding a varying degree of publications, for example, electron scanning microscope. However, the majority is expected to be related with the search results drowning out nonrelated articles. **Remarks, the data is of the date of 24/09/2024, which may not reflect on the entirety of the year 2024**. In total, 365 data entries were obtained and accumulated into a CSV file (Supporting Information) using the export function from Scopus that yield important information such as “Authors”, “Author full names”, “Author(s) ID”, “Title”, “Year”, “Source title”, “Volume”, “Issue”, “Art. No.”, “Page start”, “Page end”, “Page count”, “Cited by”, “DOI”, “Link”, “Affiliations”, “Authors with affiliations”, “Abstract”, “Author Keywords”, “Index Keywords”, “Correspondence Address”, “Editors”, “Publisher”, “ISSN”, “ISBN”, “CODEN”, “PubMed ID”, “Language of Original Document”, “Abbreviated Source Title”, “Document Type”, “Publication Stage”, “Open Access”, “Source”, and “EID”. Overall, the necessary information for analysis is the year, source title (publisher name), abstract, index keywords (keywords assigned by publishers), and correspondence address (address of corresponding author). Note, index keywords are often set by the publisher, which provides clearer searchability and categorization. Additionally, the CiteScore, which is a Scopus metric to determine the impact of a publisher, is added to the data set via Scopus Sources. Even though some experts warn against using CiteScore as a primary source on journal-based assessment,^20,21^ it is openly accessible to the general public and is transparent in how the metric is calculated, thus, this study uses this metric to determine journal impact.

### Data Preprocessing—Algorithm and Evaluation

Once the data set has been curated, preprocessing and cleaning is then done to ensure the reduction of redundant information. For the text data in this study, this is mostly done with the removal of stopwords. Stopwords are a set of words with little meaning that do not contribute to the knowledge gained (e.g., pronouns and transition words). Some of these words include “a”, “an”, “he”, etc. The stopwords used in this study is obtained from the NLTK (Natural Language Toolkit) library in Python.^22^ Furthermore, additional stopwords and special characters were added to the list of official stopwords. The full list of stopwords in the Python scraping file, coding, and Scopus data can be found on the author’s GitHub (https://github.com/Demodesu/Data-Mining-Into-Machine-Learning-Aided-2D-Material-Research-In-Electrochemical-Application). Another step in cleaning the data is the abstracts found in the Scopus database, which contains copyright markers and publisher information. This information was then removed from the abstract text for all data entries prior to analysis.

In data mining, two primary goals are considered when choosing the task, which is predictive versus descriptive.^16^ Both methods aim to extract knowledge from a given set of data; however, there are certain differences in their approach. Predictive data mining, often termed supervised data mining, involves predicting a predefined target variable based on input features. In contrast, descriptive data mining, also known as unsupervised data mining, does not aim to predict a specific target; rather, it focuses on identifying patterns and extracting insights from large data sets. This study primarily utilizes descriptive data mining, where the focus is on explaining patterns and extracting knowledge from the data. Two main methods are utilized to data mine: (1) frequency analysis and (2) network graph analysis. Frequency analysis is performed using word clouds, bar plots, pie charts, and heat maps. The word cloud is generated using a python library called “wordcloud” while the bar plots and pie charts are generated using another library called “matplotlib”. The main usage for word clouds is to visualize large quantities of frequent words that occur in the keyword section of publications. This method was chosen, as it is an easily understood format to convey and communicate data analysis. Additionally, this method can reveal the most occurring word for trend analysis, themes, and topics (e.g., “capacitance”, “current density”, “resistance” points to a theme on electrochemical properties), and even outliers that one would not expect from a certain topic. Pie charts are used for keyword and year analysis, in which the top 10 keywords found in the respective year can be found in the pie chart. Heat maps are used to represent data via two or more axis labels coupled with their respective frequencies, which is employed by the “seaborn” library in Python. This gives a comparison between interested variables and can even show trends for certain axis labels. Compared with frequency analysis, network graph analysis can show connections present in text data. Co-occurrence networks are a type of network graph that connects words that frequently appear together. The words are obtained from the abstracts of the article in which an occurrence of both words in a text is counted as a word pair. The network is then represented as nodes (words) and edges (connections) on graphs. This gives insight into themes between words and cluster related topics.

In the further sections, key points and keywords are extracted from the data mining of Scopus data, which will be analyzed. From data analysis, it was discovered that 2 main classifications of publications for the keywords 2d OR ″two dimen*″ OR ″2 dimen*″ AND material* AND “machine learn*” AND elec* are present: (1) 2D materials for improvement of machine learning (e.g., enhance computational power or speed, lower energy consumption of machine learning) and (2) Machine learning for improvement of 2D materials (e.g., optimization of parameters, discovery of new materials). This study incorporates both perspectives, as the separation of these publications is a complex task. In further sections, the extracted data are represented and explored.

## Background Study of 2D Materials

The properties of materials can change significantly when their dimensions are reduced to the nanoscale, enhancing aspects such as electrical conductivity, chemical reactivity, mechanical strength, and optical behavior.^23^ Nanomaterials are generally classified based on their dimensionality into 0D (zero-dimensional), 1D (one-dimensional), and 2D (two-dimensional) material.^24^ 2D materials are characterized by having one dimension, typically, the thickness, reduced to just a few nanometers or less.^25^ In these materials, electrons can move freely within the two-dimensional plane but their movement in the third dimension is confined.^26^ 2D materials can be broadly divided into two categories based on their original crystal structure: layered 2D materials and nonlayered 2D materials.^27^ Layered 2D materials, such as graphene and transition metal dichalcogenides (TMDs), have strong in-plane chemical bonds within their atomic layers, while the layers themselves are stacked together through weak van der Waals forces in their bulk form.^28^ In contrast, nonlayered 2D materials, such as MXenes and transition metal oxides (TMOs), originate from bulk materials with strong three-dimensional chemical bonds.^27^ When these materials are transformed into their 2D form, they expose unsaturated surface atoms, which enhances their reactivity and provides additional flexibility for forming heterostructures and tuning their structural configurations.^29^ The first modern 2D material, graphene, was discovered in 2004 by a team led by Andre Geim and Kostya Novoselov.^30^ Graphene is composed of a single layer of carbon atoms arranged in a honeycomb (hexagonal) lattice, bonded through strong sp^2^ hybridization.^31^ The researchers isolated graphene from bulk graphite using mechanical exfoliation, commonly known as the Scotch tape method, to peel atomically thin layers and transfer them onto a silicon wafer substrate coated with a thin layer of SiO_2_.^30^ This ground-breaking discovery marked a significant advancement in materials science, proving that stable, single-layer, and few-layer materials can exist while exhibiting extraordinary and technologically valuable properties. Graphene is renowned for its unmatched thermal conductivity, optical transparency, and outstanding electrical conductivity.^32–34^ These properties position graphene as a highly promising material for a wide range of applications, particularly in electrochemical energy storage devices.^35^ Since then, the success of graphene has inspired the exploration of a wide variety of 2D materials, with over a hundred now being extensively studied for their unique characteristics and applications.^36^ Among the many emerging families of 2D materials, transition metal dichalcogenides (TMDs) have gained recognition as some of the most promising layered 2D materials. Often regarded as potential alternatives to graphene, TMDs have paved the way for an extended lineage of research and development in the field.^37^ 2D transition metal dichalcogenides (TMDs) have garnered significant attention due to their natural abundance and a wide range of exceptional properties. These materials are inherently thin, transparent, and flexible.^25^ Unlike graphene, which has a zero band gap, TMDs are naturally semiconducting, making them particularly appealing for a variety of applications.^38^ Their unique properties position TMDs not only as promising candidates for energy storage systems^39^ but also as ideal materials for nanoelectronics and optoelectronic devices.^40,41^ Although both graphene and TMDs are classified as layered 2D materials, their structures differ significantly. Graphene consists of a single atomic layer of carbon atoms arranged in a hexagonal lattice. In contrast, TMDs are composed of a transition metal (M) layer sandwiched between two layers of chalcogen atoms (X), conforming to the stoichiometric formula MX_2_.^42,43^ Within each layer, the atoms are bonded by strong covalent bonds, while the layers themselves are held together by weak van der Waals (VDW) interactions.^44^ There are approximately 50 combinations of TMDs with the general formula MX_2_, where M represents transition metals from groups 4 to 10 (commonly V, Nb, Ta, Mo, W, Re),^45^ and X is a chalcogen atom, typically sulfur (S), selenium (Se), or tellurium (Te).^46^ This extensive variety of combinations results in a broad spectrum of properties across the TMD family, ranging from insulators like HfS_2_, to semiconductors such as MoS_2_, semimetals like TiSe_2_, and even true metals like NbSe_2_, which can exhibit superconductivity at low temperatures.^47^ The diverse properties and potential applications of TMDs have sparked considerable experimental research. The synthesis method of 2D nanosheets is a critical factor in experimental research. Graphene and 2D TMDs can be synthesized using similar techniques, as their layers are held together by weak van der Waals forces. Common methods for producing these materials include mechanical exfoliation, liquid-phase exfoliation, chemical vapor deposition (CVD), and solvothermal or hydrothermal techniques.^48^ In contrast, such conventional methods are rarely used or unsuitable for fabricating nonlayered 2D materials like MXenes, which require distinct synthesis approaches to produce 2D nanosheets. MXene is an emerging class of 2D materials first discovered in 2011, with Ti_3_C_2_T_X_ being the inaugural member of the family.^49^ MXenes are composed of transition metal carbides, carbonitrides, and nitrides, described by the general formula M_*n* + 1_X*_n_* T*_X_*, where M represents transition metals such as Sc, Ti, V, and Cr, X denotes carbon and/or nitrogen atoms, and T_X_ refers to surface functional groups like −O, −OH, or −F. The parameter n can take values of 1, 2, or 3.^50^ MXenes are typically synthesized from layered MAX-phase precursors (M_*n* + 1_AX*_n_*) through selective chemical etching, which removes the A-layer (commonly a group 13 or 14 element, such as Al or Si).^51^ This process usually involves the use of strong acidic etching solutions containing fluoride ions (e.g., hydrofluoric acid, HF).^52^ Following etching, conventional techniques such as intercalation and sonication are employed to delaminate the stacked MXene layers into single or few-layer nanosheets.^53^ MXene nanosheets stand out among typical 2D nanomaterials due to their exceptional properties, including hydrophilicity, high conductivity, and catalytic activity.^23^ These characteristics position MXenes as optimal candidates for a wide range of applications, from energy storage to photocatalysis and biomedicine.^53^ Since their discovery, more than 40 distinct MXene compositions have been synthesized, with computational design and experimental advancements promising the potential for many more.^54^ Furthermore, the creation of composites combining 2D materials like graphene, TMDs, and MXenes has unlocked diverse properties and expanded the potential applications of these materials. This diversity has driven extensive experimental research, engaging countless researchers and dedicating thousands of hours to exploration. However, given the vast number of possible combinations and their immense potential, computational studies have become increasingly vital to advancing our understanding of 2D materials and guiding future innovations.

## Background Study of 2D Materials in Machine Learning

Machine learning usage with 2D materials has increased prolifically in recent years due to the advancement of computational power and tools available for both private and public use (e.g., Scikit-learn, MATLAB). These tools are then utilized to solve issues related to 2D materials. Some of these issues include the optimization of the 2D material structure, discovering new insights from previous data, and predicting/forecasting the parameters or trends. Our group was the first researchers in Thailand who heavily incorporated data science and machine learning into 2D material electrochemical research, paving the ground for advancement in so-called “Electrochemical Intelligent” in Thailand. Their works include utilizing neural networks to discover optimal doping conditions of graphene supercapacitors by Chenwittayakhachon et al.,^15^ utilizing data science with graphene supercapacitor data by Jitapunkul et al.,^55^ researching how machine learning can be used in the optimization of liquid exfoliation of 2D materials,^48^ and utilizing machine learning in the optimization of gold-decorated graphene for use as a catalyst in hydrogen evolution.^56^ However, these studies are just a small part of the research by the whole scientific community. It is then a pressing matter that a macroscopic overview of the scientific community is done. This leads to utilizing data mining and KDD to expand the knowledge base of both the authors and readers alike.

## Current Progress in 2D Materials with Machine Learning Using Knowledge Discovery in Databases (KDD)

A heat map of the number of publications per year and country can be found in Figure 2. From the results, China, America, Germany, and India are the leading countries for machine learning in 2D materials for electrochemical use, with 110, 77, 31, and 24 publications, respectively. The earliest publication related to this topic first appeared in America in the year 2013, when Converse and Fullwood at Brigham Young University, America, published the title “Enhancing nanoscale SEM image segmentation and reconstruction with crystallographic orientation data and machine learning”.^57^ They proposed the enhancement of scanning electron microscopy imagery through the use of crystallographic orientation data and machine learning. The material they used was conductive nanocomposites composed of nickel nanostrands suspended within an epoxy matrix. They discovered that the method is fairly accurate (71.9% for 2D algorithms and 62.4% for 3D algorithms). Furthermore, it is demonstrated that the algorithm can predict gaps between nanostrands, which can be difficult to detect with only scanning electron microscopy. Even though this research is found by a search query, it demonstrates the beginning of 2D materials and machine learning. Obviously, the number of publications has significantly increased since the year 2019. This marked the rise of machine learning and artificial intelligence in scientific research with the case of the social distancing measure, which shifted some scientists to progress their research from a laboratory approach to a computational approach during the “Covid19”. Additionally, computational power has increased significantly, enabling machine learning to be done with the graphic processing unit (GPU) of the computer (e.g., tensorflow^58^), leading to faster computation; thus, more machine learning usage.^59^ Another interesting observation is that America caught onto the trend faster (starting in 2013), but China released more publications in the later years (2022 onward), resulting in more publications. The heat map also suggests a steadily increasing trend that has not hit a peak. This means that all countries are slowly starting to catch up with increasing numbers of publications per year. Upcoming countries that are catching up to the trend are Australia, Canada, Germany, India, Korea, the United Kingdom, and Singapore.

**Figure 2:**
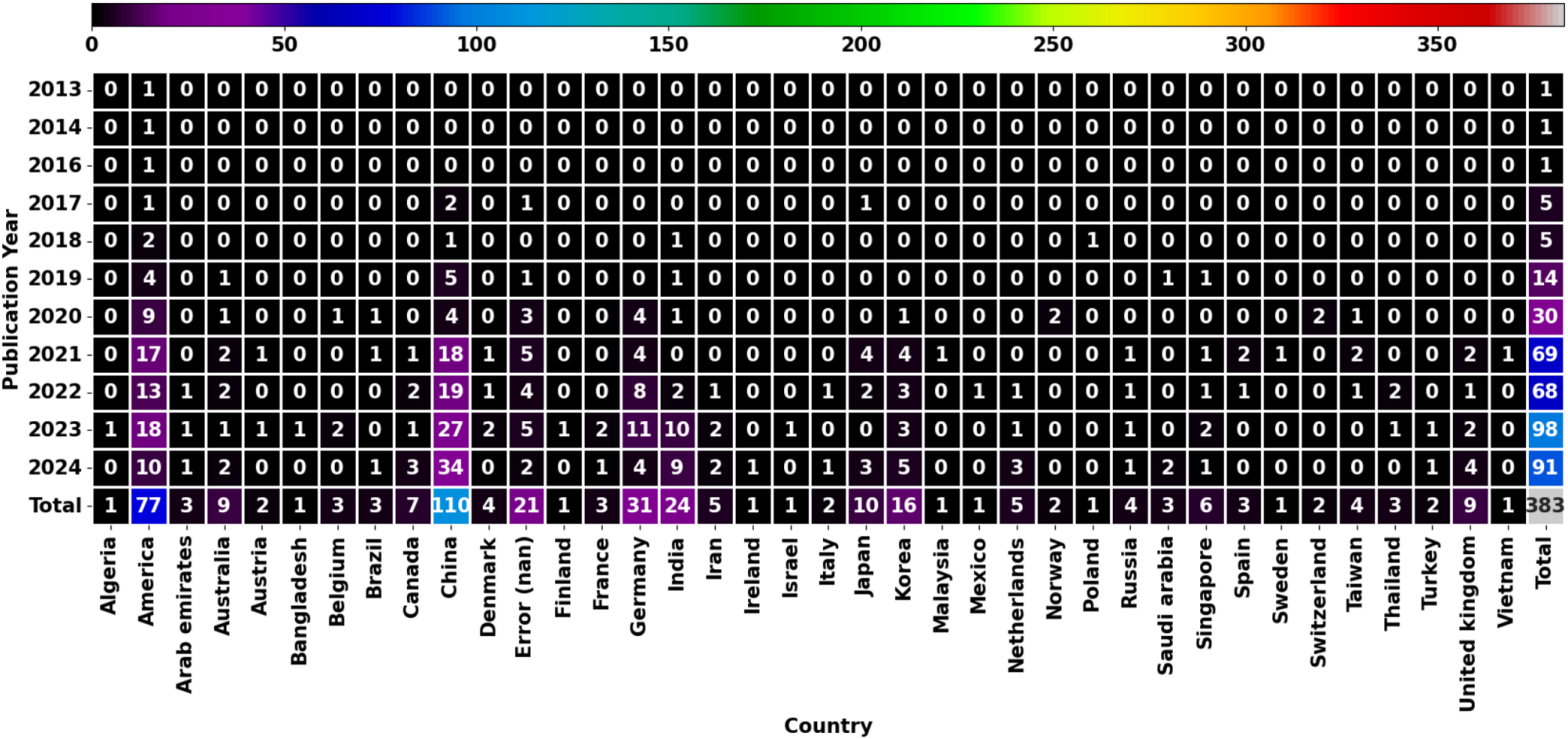
Heat map of year, country, and amount of publication, which shows leading countries concerning the topic of 2D materials, machine learning, and elec* related topics (as of 24/09/2024).

Although publication amount is a useful metric in determining the trend-setter, it does not account for the impact of the journal, which shows the leading publication that brings innovation to that field. In Figure 3, the heat map of countries with their publication amount and highest citation paper can be seen. Ranking the top three publications, which are in Germany, China, and India (391, 352, and 268 citations respectively). Germany published a paper titled “Exceptional piezoelectricity, high thermal conductivity and stiffness and promising photocatalysis in two-dimensional MoSi_2_N_4_ family confirmed by first-principles by Mortazavi et al. in 2021.^60^ Here, they explored various properties (e.g., thermal conductivity and photocatalytic ability) of a novel 2D material, MA_2_Z_4_ (M = Cr, Mo, W; A = Si, Ge; Z = N, P). Machine learning and first-principles calculations utilizing density functional theory (DFT) were done to reveal the inter-atomic potentials to examine the properties of complex 2D materials. They achieved this by training a moment tensor potential (MTP) on ab initio molecular dynamics data. The study discovered that the material can compete with popular materials such as TMDs and graphene for various applications. China published their paper titled “Simultaneously Achieving High Activity and Selectivity toward Two-Electron O_2_ Electroreduction: The Power of Single-Atom Catalysts in 2019 by Guo et al.,^61^ which explored catalysts for H_2_O_2_ production. They utilized DFTs to initially screen 210 single-atom catalysts (SACs) for their stability, selectivity, and activity under acidic conditions. The results yielded 31 highly promising SACs for H_2_O_2_ production, with 7 having high activity in acidic conditions. Overall, a single Zn atom centered in phthalocyanine (Zn@Pc-N_4_) showed remarkable activity (0.15 V overpotential). They then used machine learning to perform feature importance analysis and explained the origin of the selectivity and activity performance between the structure and catalysts. For adsorption, they utilized multiple linear regressions to unlock the weights of features (electronic properties and bonding characteristics). Additionally, they utilized random forests to obtain feature importance of features for chemical properties of central atoms and the chemical environment and discovered that the adjusted electron numbers of the d/p orbital and the oxide formation enthalpy were the most important features. Lastly, India published their paper titled “Machine-Learning-Assisted Accurate Band Gap Predictions of Functionalized MXene in 2018 by Rajan et al., which aims to reduce the time in DFT simulations for band gap of MXenes using machine learning. They utilized kernel ridge regression, support vector machines, Gaussian process, and bootstrap aggregating (bagging) algorithms trained on MXene properties (e.g., boiling and melting points, atomic radii, bond lengths).^62^ The results concluded that the Gaussian process model achieved the lowest root mean squared error of 0.14 eV in a short fraction of time. Other than top citations, the number of citations that a country has produced can show the leading innovators of the field. Astonishingly, two countries have over 1000 citations, leading to the research space. China leads machine learning in the 2D materials field with 1692 (27.21%) overall citations, which is a quarter of publications, and America comes out next with 1260 overall citations (20.26%).

**Figure 3:**
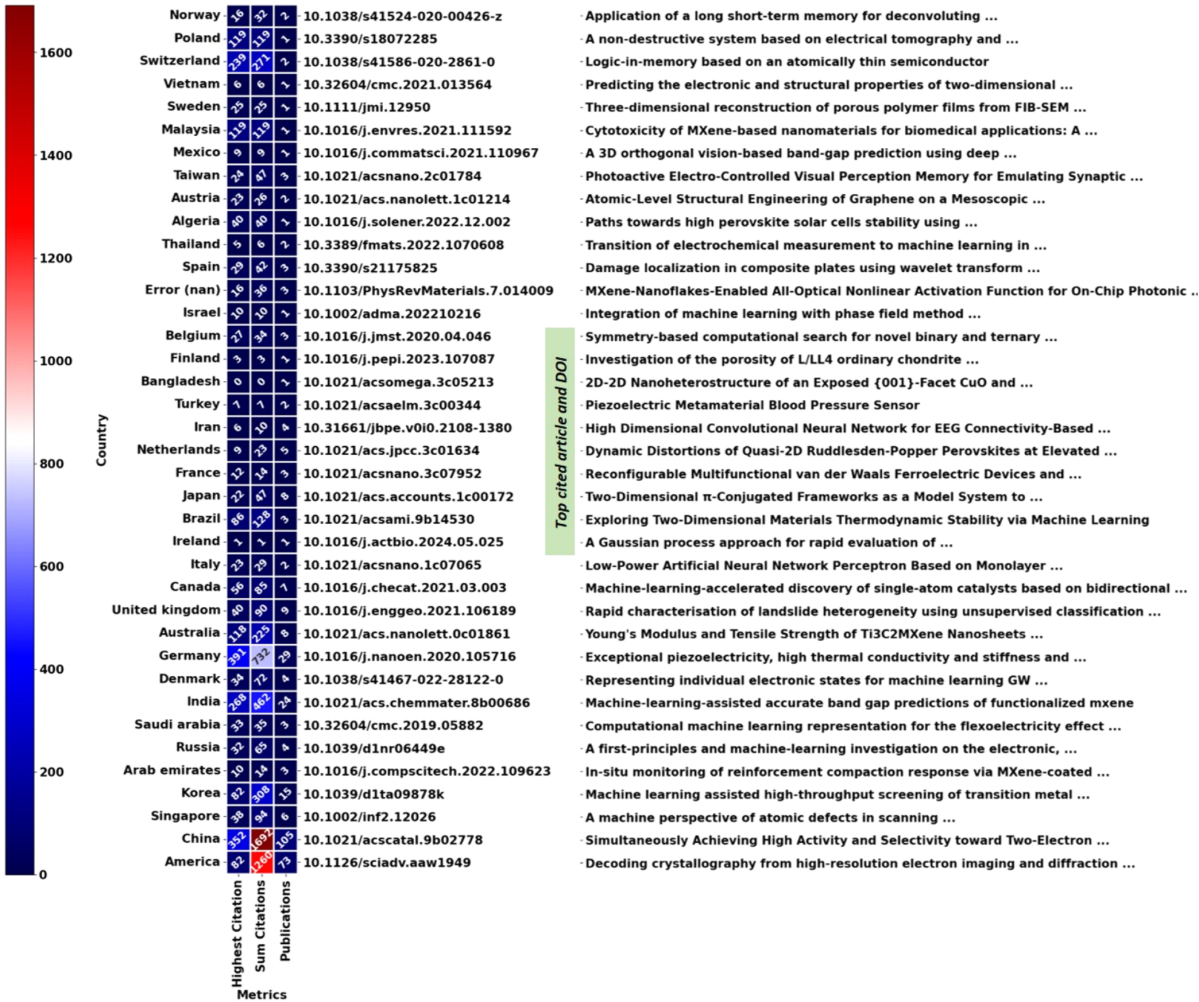
The heat map of citation metrics (e.g., highest, sum, and amount), country, title, and DOI of the highest cited papers, showing insight such as country with the highest number of citations, highest cited paper, etc. (as of 24/09/2024).

Another metric, CiteScore, is obtained from Scopus to enhance the clarity in which countries bring leading innovation to the 2D materials field using machine learning. By studying this metric, researchers can get insight into interesting publications published in leading journals before trends catch on. From Figure 4, it can be seen that America and Switzerland now lead the field in their top CiteScore journal, Nature, with a CiteScore of 90. The leading publication found for Switzerland is “Logic-in-memory based on an atomically thin semiconductor”^63^ by Migliato Marega et al. in 2020. They propose using a 2D material in the class of TMDs called molybdenum disulfide (MoS_2_) to develop atomically thin semiconductors for advanced low-power electronics. They hinted at the use of this new material with energy-efficient electronic hardware applications incorporated with machine learning in the future. For America, the publication “Moiré synaptic transistor with room-temperature neuromorphic functionality”^64^ by Yan et al. in 2023 was published in the renowned journal Nature. They utilized 2D materials such as a graphene/hexagonal boron nitride bilayer heterostructure to make a room-temperature synaptic transistor, which revolutionizes artificial intelligence and machine learning by efficient hardware upgrades. Another high CiteScore publication comes from China in the Nature Nanotechnology journal. The publication comes from Ning et al. in 2023 and is titled “An in-memory computing architecture based on a duplex two-dimensional material structure for in situ machine learning”.^65^ Here, they used an atomically thin MoS_2_ material transistor to create an energy-efficient device for in-memory computing. They tested the device on a complex machine-learning task and achieved 99.86% accuracy. Although most of the research works mentioned are not machine learning based, they all mention the utilization of 2D materials for more energy-efficient and powerful machine learning (2D materials for the improvement of machine learning). Looking deeper into more countries with high CiteScore (40+), the top publications of each country are highlighted as follows: (1) Singapore utilized machine learning and first-principles calculations to validate their experiments of spin-polarized graphene.^66^ (2) Korea used CuInP_2_S_6_ (CIPS), a novel 2D material, to generate true random numbers, which is useful in machine learning applications.^67^ (3) Germany utilized machine learning trained on density functional theory data (DFT) to predict flexoelectric energy conversion in 2D bilayers.^68^ (4) United Kingdom used machine learning, specifically generative adversarial networks (GANs), which are typically used in image generation, to generate 3D mesostructural data of materials from 2D imagery.^69^ (5) Isreal demonstrated the use of MXenes in optical computing via incorporation of MXenes into on-chip neural networks.^70^ These publications highlight the emergence of 2D materials for machine learning (Korea and Isreal) and machine learning for 2D materials (Singapore, Germany, and the United Kingdom).

**Figure 4:**
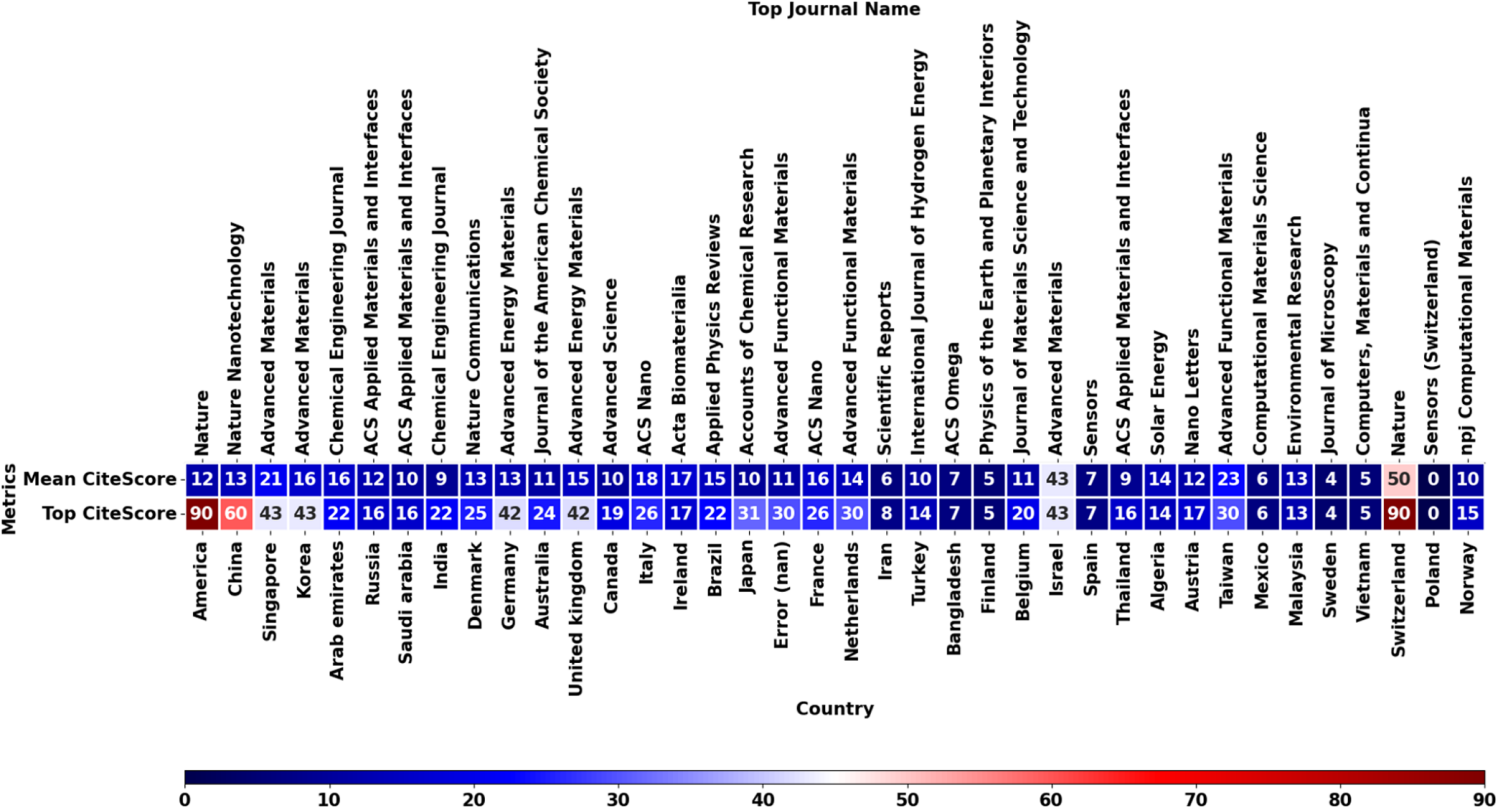
Heat map of CiteScore, published journals, country, and top publishers showing the countries that published in leading journals (as of 24/09/2024).

To gain further insight into the trends of 2D materials and machine learning, a word cloud of the most frequent words in the index keywords can be used. The results can be seen in Figure 5, which counts the number of whole words (i.e., the entire keyword). With new insight in frequent words, trends can be examined with a deeper dive into the subject. Additionally, plural words are combined with their singular counterparts. Remarks, the larger the word in the word cloud, the more frequent it appears in publications. For clearer indication, the 10 most frequent words are listed in the following order: (1) density functional theory (116), (2) transition metals (45), (3) learning systems (44), (4) human (40), (5) learning algorithm (38), (6) energy gap (38), (7) graphene (38), (8) forecasting (35), (9) electronic properties (35), and (10) layered semiconductors (34). From the whole words word cloud (Figure 5), the most frequent keyword is density functional theory (DFT). DFTs is a computationally effective and relatively accurate method of approximating many-body electron simulations.^71^ This allows the simulation of material properties at the atomic scale, which is used in materials science to understand the relationships between structure and composition of materials.^72^ Other possible related keywords to DFTs are electronic properties and molecular dynamics, which are also present in top publications (e.g., piezoelectric property and band gap). Overall, a large number of studies have found utilized DFT to generate a large batch of ab initio data as their main source of data for model training, after which the model is used to discover new properties or materials. In terms of applications, frequent words such as catalysts,^73,74^ hydrogen evolution,^75–77^ semiconductors,^78,79^ and electrodes^80,81^ were found. This highlights the leading use of 2D materials coupled with machine learning in these fields. Not only is machine learning used to discover new materials, but it can also be used in the optimization of existing materials. For example, the word cloud highlights keywords such as graphene,^13,14,82^ transition metal dichalcogenides (TMDs),^83–85^ and MXenes.^86–88^ Another interesting material is boron nitrides, which is a unique 2D material with a hexagonal shape similar to that of graphene. These materials can be optimized for thermal conductivity, electronic properties, (e.g., capacitance and energy gap), optical properties, and more. Additionally, the most common models used for machine learning can be found in the word cloud. The word cloud shows several usages of machine-learning models, such as regression analysis, predictions, and forecasts. Popular models include a convolutional neural network,^89^ artificial neural network,^90,91^ and decision tree.^92^ Furthermore, some keywords that do not appear in the 10 most frequent keywords but are of interest are the synthesis method called chemical vapor deposition and the materials used, which are carbides, perovskites, sulfur compounds, tungsten compounds, selenium compounds, and molybdenum compounds. This highlights the most frequent synthesis methods and materials used, which can guide researchers in selection and fabrication. In further sections, these keywords will serve as the basis for a more in-depth literature review of the findings.

**Figure 5:**
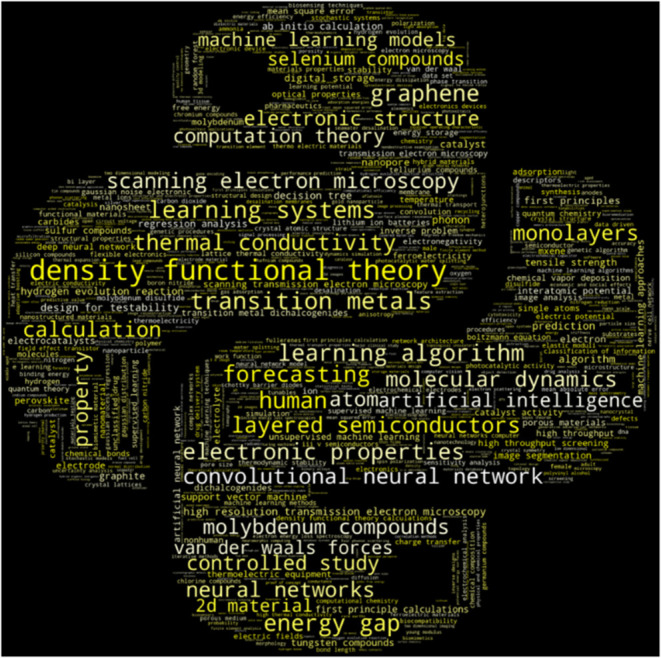
Word cloud of the most frequent words in the topic of 2D materials, machine learning, and elec* related topics.

Year and keyword analyses are done to gain further insight into the trend of 2D materials (Figure 6). The pie chart includes the year of publications with surrounding keywords. The keywords are the top 10 most commonly found keywords in that year’s publications. **Remarks**, only the recent years (2019+) will be shown, as the large quantity of years and keywords reduce the readability of the graph. In 2013, only 15 keywords were found. This was the study done by Matthew I. Converse and David T. Fullwood mentioned in the beginning of the section, where the country and year heat map is found, where they utilized machine learning to improve SEM imagery. Keywords such as scanning electron microscopy and image segmentation can be found. In 2016–2018, the trends of utilizing machine learning steadily increased, with keywords such as transition metal dichalcogenides and genetic algorithm appearing. This marks the first appearance of a specific model or algorithm, the genetic algorithm, being a part of the main keywords. Additionally, transition metal dichalcogenides caught on early, appearing in 2018. In recent years (2019+), the trend has seen exponential growth, growing from 231 keywords to 1838. Materials used were graphite, graphene, and transition metal dichalcogenides. These materials have seen a range of usage, ranging from catalysts to semiconductors. The most predicted property seems to be the energy gap, which is present in semiconductor research. Interestingly, density functional theory was popular all the way from 2019 to 2024, remaining in the top 10 keywords for 5 years. Therefore, a combination of density functional theory and machine learning is likely to continue to be used in the future. Neural networks have seen extensive use in the year 2021, likely due to their high accuracy, but lack in interpretability due to it being a “black box” model.^93^ Lastly, the trends of an increasing number of keywords can be seen from Figure 6g. This plot shows the total number of keywords found in each pie chart (year) and plots it compared to their respective year. Although this is not an accurate way to assume the rise of machine learning in 2D materials for electrochemical use, it can be another metric in determining the rising trends. Judging from the graph in Figure 6g, it is assumed that an annual increase of roughly 200 to 300 keywords can be found in the year 2025. Additionally, Density Functional Theory (DFT), catalysts, and prediction of electronic properties research is assumed to continue in further years. These data show that there is an ever-increasing interest in machine learning usage in 2D materials, which suggests that this field will continue to rise well into the future.

**Figure 6:**
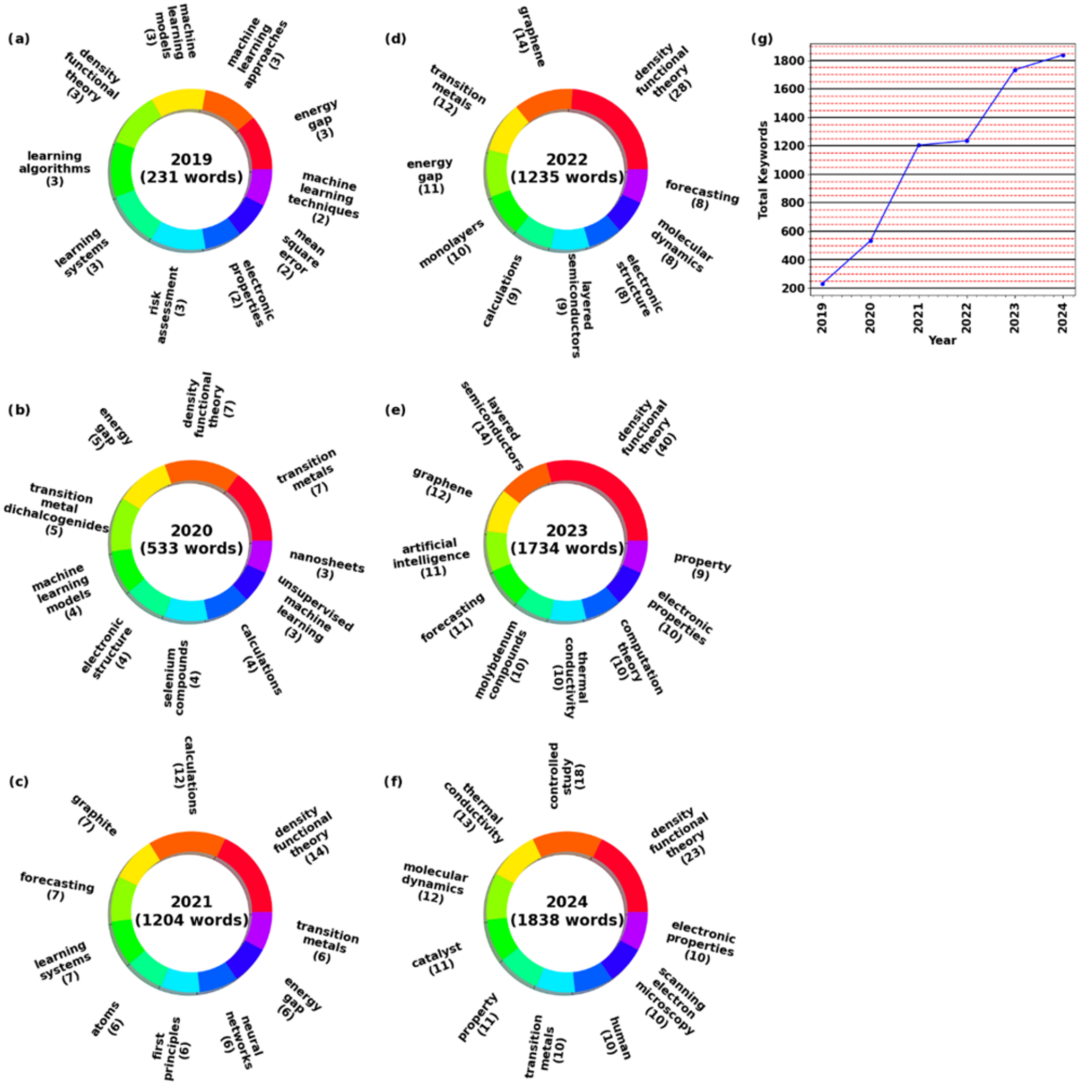
Year and keyword pie chart showing the top 10 most frequent words found in the keywords for the year (a) 2019, (b) 2020, (c) 2021, (d) 2022, (e) 2023, (f) 2024, and (g) the increasing keywords in each year.

Another useful metric to observe trends and connections is via co-occurrence networks. Co-occurrence networks group words into pairs based on how often they appear jointly. The connections can then be represented by various graphs, in this case, a spring network graph. However, due to the sheer number of connections between words that appear, only connections that have 8 or more occurrences will be reported in this study. Additionally, the words that correspond to the index of the spring network can be seen in the table below. The connections (edges) of the graph are colored and widened based on the frequency of occurrence. In the spring network (Figure 7), low connections and clusters between words can be easily seen. Additionally, the corresponding index and words can be seen in Figure 8. For example, the word chain water (184)–splitting (185)–photocatalytic (186) shows the application of photocatalysis being largely focused on water splitting.^60,94–97^ From the clusters, the largest node is the optical (21)–electronic (20)–dimensional (13)–mechanical (34)–thermal (78)–electrical (77)–physical (18)–chemical (19)–properties (22)–transport (88) node, in which the central node, properties (22), connect up to 9 other nodes. This shows the main properties of studies when applying machine learning to 2D materials. Medium word nodes (>5 word connections) show relationships worth noting. For example, the electrochemical (163)–free (110)–storage (106)–energy (40)–conversion (76)–formation (39) shows the main focus of electrochemical studies, which are mainly focused on energy conversion and storage.^98–102^ Lastly, the largest cluster found in the middle is the dichalcogenide (16)–lattice (158)–dft (9)–flexible (32)–initio (116)–stability (79)–scale (3)–chemical (19)–calculation (10)–single (129)–metal (15)–ab (115)–sac (132)–atom (130)–transition (14)–catalyst (131)–conductivity (90)–property (174)–electronic (20)–tmd (17)–computational (189)–boltzmann (190)–properties (22)–vapor (27)–low (82)–band (94)–nitride (128)–first (35)–carbide (178)–frequency (103)–large (2)–gap (96)–electrical (77)–physical (18)–transport (88)–atomic (170)–three (31)–functional (7)–optical (21)–equation (191)–carbon (127)–theory (8)–dimensional (13)–structure (95)–thermal (78)–mechanical (34)–semiconductor (57)–deposition (28)–density (6)–device (58)–edge (187)–alignment (188)–crystal (97)–cost (83)–principle (36)–dynamical (93) cluster. This shows the highly complex interconnection of topics published by researchers in this space.

**Figure 7:**
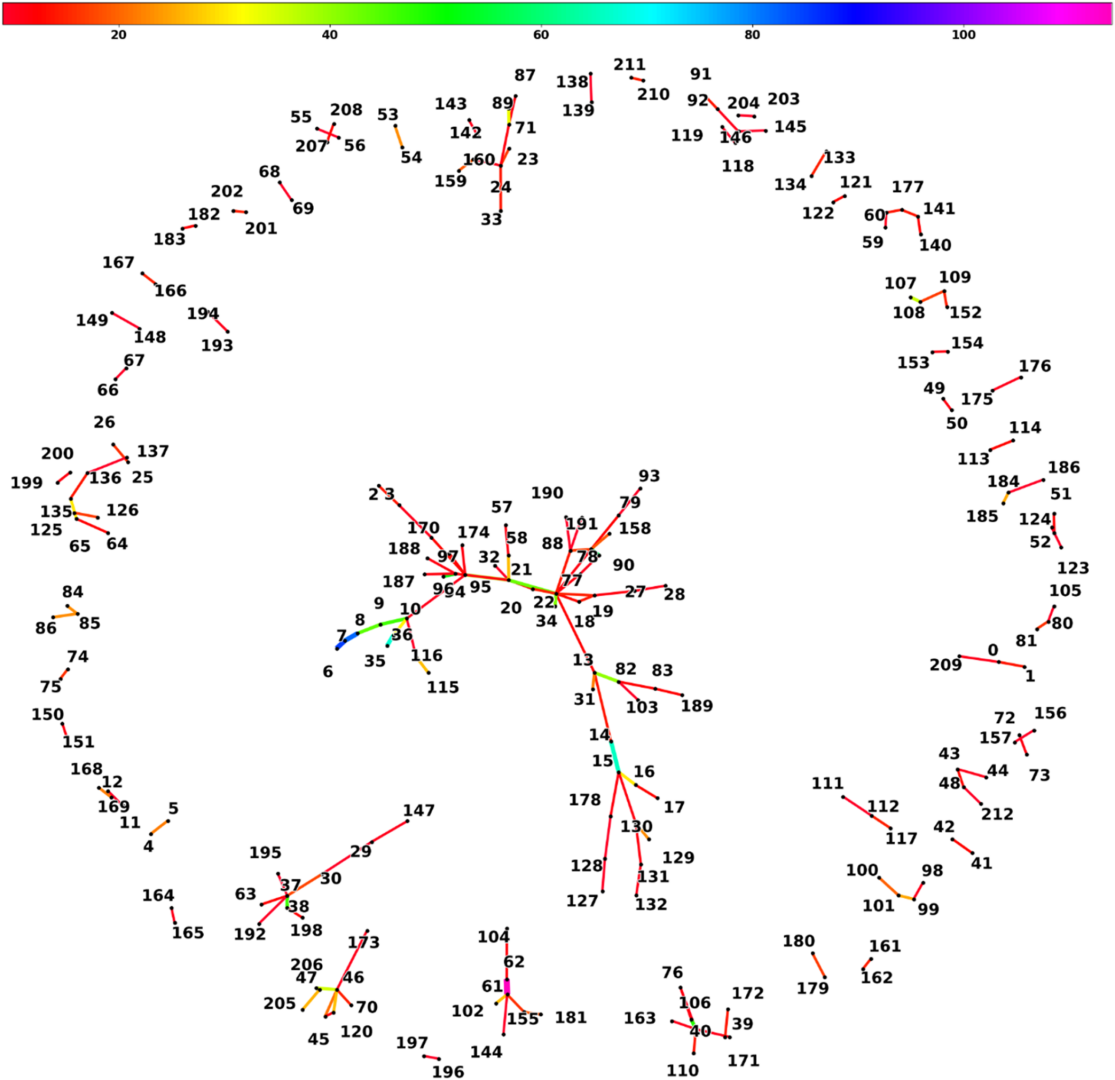
Spring co-occurrence network of abstract text showing clusters of connected keywords; the color of connection shows how many times the words co-occur.

**Figure 8:**
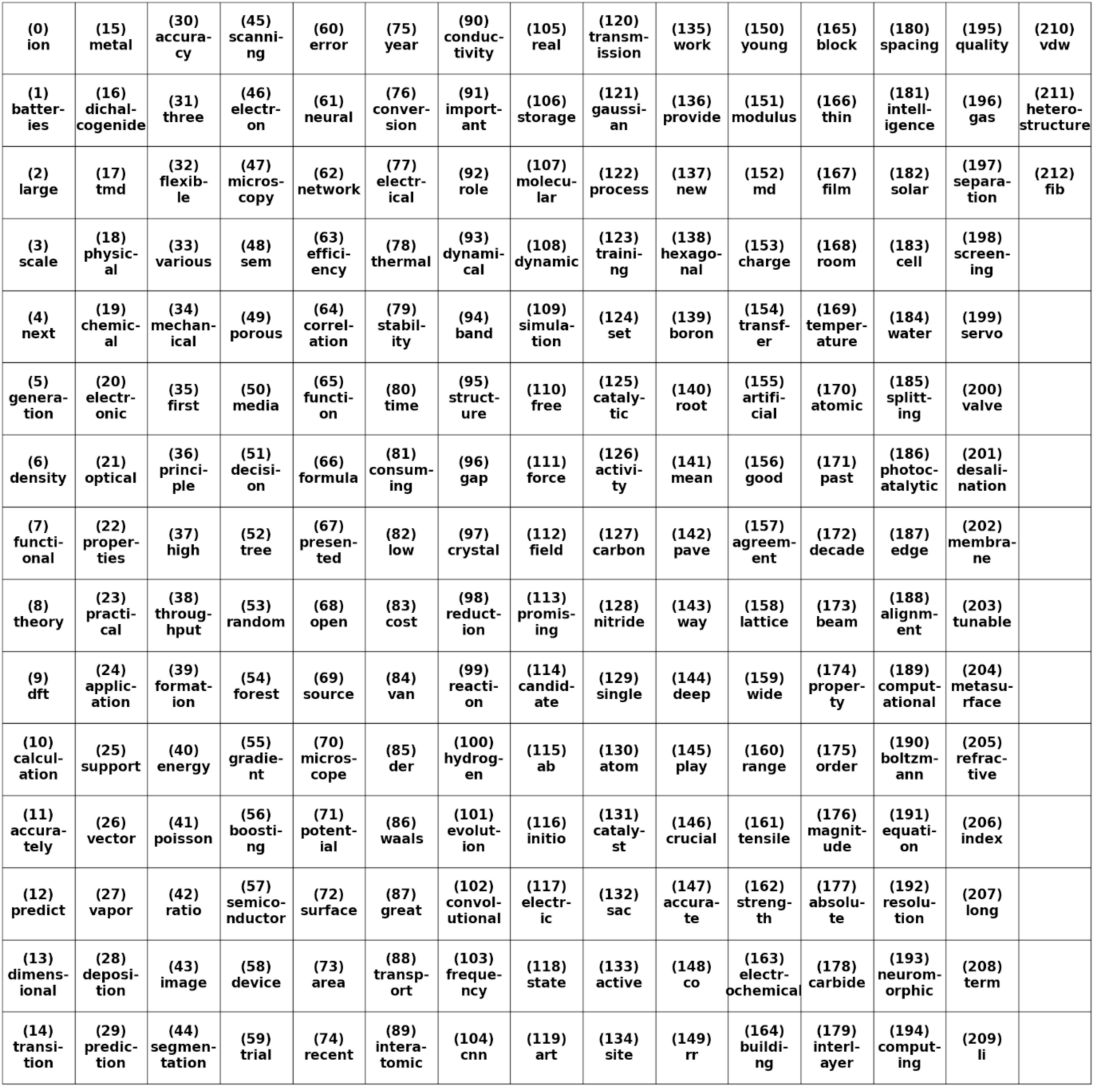
Spring co-occurrence network of abstract text word table; each index represents the corresponding word in the spring network.

Overall, this section obtains keywords and deeper topics from KDD, allowing for a more defined search. In further sections, keywords obtained from this section will be analyzed. Hence, these sections will prove as a deep dive into the literature review of the findings. However, a limited number of data is available from the broad search query used in this study (2d OR ″two dimen*″ OR ″2 dimen*″ AND material* AND “machine learn*” AND elec*), therefore, additional publications are manually curated from various sources using a mix of keywords while still remaining in the topic of 2D materials, machine learning, and electrochemistry. Consequently, this allows screening of topics not related to electrochemistry as the elec* keyword searches for all things related to elec* (e.g., electronics, electricity, and electrons). This search query is not accumulated into a CSV, but is referenced in their respective sections. The main keywords (obtained from word clouds, network graphs, analysis) present in this search are as follows: **density functional theory**, **energy conversion**, **semiconductor**, **catalysts**, **transition metal dichalcogenides**, **MXenes**, **graphene**, **photocatalytic**, and **hydrogen evolution**. These keywords will be the focus in the analysis of further sections.

## Machine Learning with Experimental and Theoretical Data

Data for machine learning were obtained from various sources. One source of theoretical calculations is Density Functional Theory (DFT). As introduced, DFTs are computationally effective and relatively accurate methods of simulations. It is often used as a tool for data generation alongside traditional experiments.^103,104^ However, DFTs struggle with large systems^105,106^ (i.e., computational complexity) and specific errors (e.g., delocalization error^107,108^ ). To resolve this, researchers proposed using machine learning to aid DFTs in their calculations of large systems^109^ (e.g., machine learning force fields^110,111^ ) and to solve certain errors in DFTs, for example, the problem of self-interacting electrons.^112^ This has caused DFTs to progress significantly in accuracy and versatility of simulations and therefore is increasingly being used among researchers. For example, Himmet et al. utilized machine learning incorporated DFTs, in this case, machine learning force fields, to evaluate MA_2_Z_4_ (M = Ti, Zr, Hf; A = Si, Ge; Z = N, P, As, Sb, Bi^113^) family monolayers properties for use in photocatalytic water splitting.^95^ Machine learning force fields (MLFF) allows the simplified calculation of complicated vectorial forces on an atom using a representative training set of atomic environments and machine-learning models,^111^ thus, reducing time and computation of complex systems. Here, it is used to validate the thermal stability of the WSi_2_N_4_ and WGe_2_N_4_ monolayers. They then discovered that the monolayers are stable at 300 K, which is an example of utilizing these tools for identifying properties. Similarly, Jadav et al. used MLFF to aid in the calculation of material properties (thermal structural stability) of MXene materials, specifically, Hf_3_C_2_F_2_, in metal ion batteries.^114^ Their aim was the utilization of MLFF to reduce molecular dynamics simulation time when performed at ambient temperature. Not only can these methods be used for identifying properties, but also they can be used to screen a large quantity of candidates. Wang et al. successfully screened 316,505 candidates down to 11 for use in photocatalysts from the Virtual 2D Materials Database (V2DB).^94^ First, they set criteria such as a band gap of 1.23 eV ≤ *E*_g_^ML(GW)^ ≤ 3 eV, which is in the range of water splitting free energy and sunlight harvesting, and set a reduction potential of the conduction band to be higher than – 5.67 eV, with the value being lower than −4.44 eV for the valence band for screening. After which, machine learning was used to confirm and validate the predicted band gap values of the materials screened from V2DB. This reduces uncertainty as V2DB outlines error metrics for the predicted data in general, not individually. This shows the great advantage of having a tool to reduce the workload of researchers in validating the properties and synthesizing a tremendous number of samples. After the initial screening process, the obtained insight can be validated with traditional experiments to confirm their findings. This encompasses the whole cycle of discovery, synthesis, and validation, which is the ultimate goal of theoretical screening. This was done by Pradhan et al., who utilized machine learning, DFT, and experimental validation.^116^ They sequentially used DFTs to predict the electrical and structural properties of an MXene quantum dot graphene composite (PO-MXQDs/rGO) via examining the interaction of charge transfer from PO-MXQDs to rGO, and then, they used Artificial Neural Networks (ANN) with different input parameters to optimize materials compositions, concentrations, and types of electrolytes to get the best super capacitive performance (1137.5 Fg^–1^). After which, the most optimized material was then synthesized for use in flexible microsupercapacitors and asymmetric coin cell supercapacitors. Their study shows the full usage and flow of DFTs, machine learning, and traditional experiments for the advancement of research. Not only can machine learning and DFTs be used for determining candidates with appropriate properties and optimizing supercapacitors, but it can be used to screen novel batteries. In this case, Zhou et al. used machine learning and DFTs to screens potential anchoring materials in the MA_2_Z_4_ family for lithium–sulfur batteries.^117^ Lithium–sulfur batteries differ in their mechanism compared to lithium batteries in that they do not rely on ion intercalation, rather, they rely on the complicated multistep electrochemical reactions involving lithium polysulfides.^118^ However, this brings new complications, such as the shuttle effect, which can degrade the performance of the battery.^119^ Therefore, new materials, which aid in the “anchoring” of soluble polysulfide, are designed to inhibit lithium polysulfide migration, which could aid in the avoidance of the shuttle effect. First, they used DFTs to calculate the binding energy of materials in the MA_2_Z_4_ family, in which materials must exhibit adsorption energy that is more than the electrolyte adsorption energy (∼0.77 eV for 1,2-dimethoxyethane, a typical electrolyte in lithium–sulfur batteries). They discovered that MoGe_2_N_4_ and WGe_2_N_4_ had an overall binding energy of ∼0.72 to 2.67 eV, which is suitable for this application. To find which properties (e.g., electron affinity and van der Waals radius) significantly contribute to the adsorption energy, machine-learning models, specifically tree-based models, were used to determine feature importance. They discovered that the electronegativity of the Z significantly contributes to the adsorption energy, the electron affinity slightly affects, and the van der Waals radius of A has a small effect on the adsorption energy of Li_2_S_2_. This study highlights the use of machine learning in conjunction with DFTs to improve energy storage devices.

Overall, experiments go through 3 major steps: (1) data gathering/generation, (2) optimization with machine learning, and (3) validation of results. To simplify these steps for researchers, a workflow diagram (Figure 9) has been made to guide researchers wishing to incorporate DFTs and machine learning in 2D materials research. The workflow walks through the hypothesis down to the gathering of data from various sources, then, machine learning is utilized to optimize, screen, or discover new materials. Finally, the results are then validated with literature or synthesis experiments and compared. Finally, the results are concluded or the process is repeated again if conclusions cannot be drawn.

**Figure 9:**
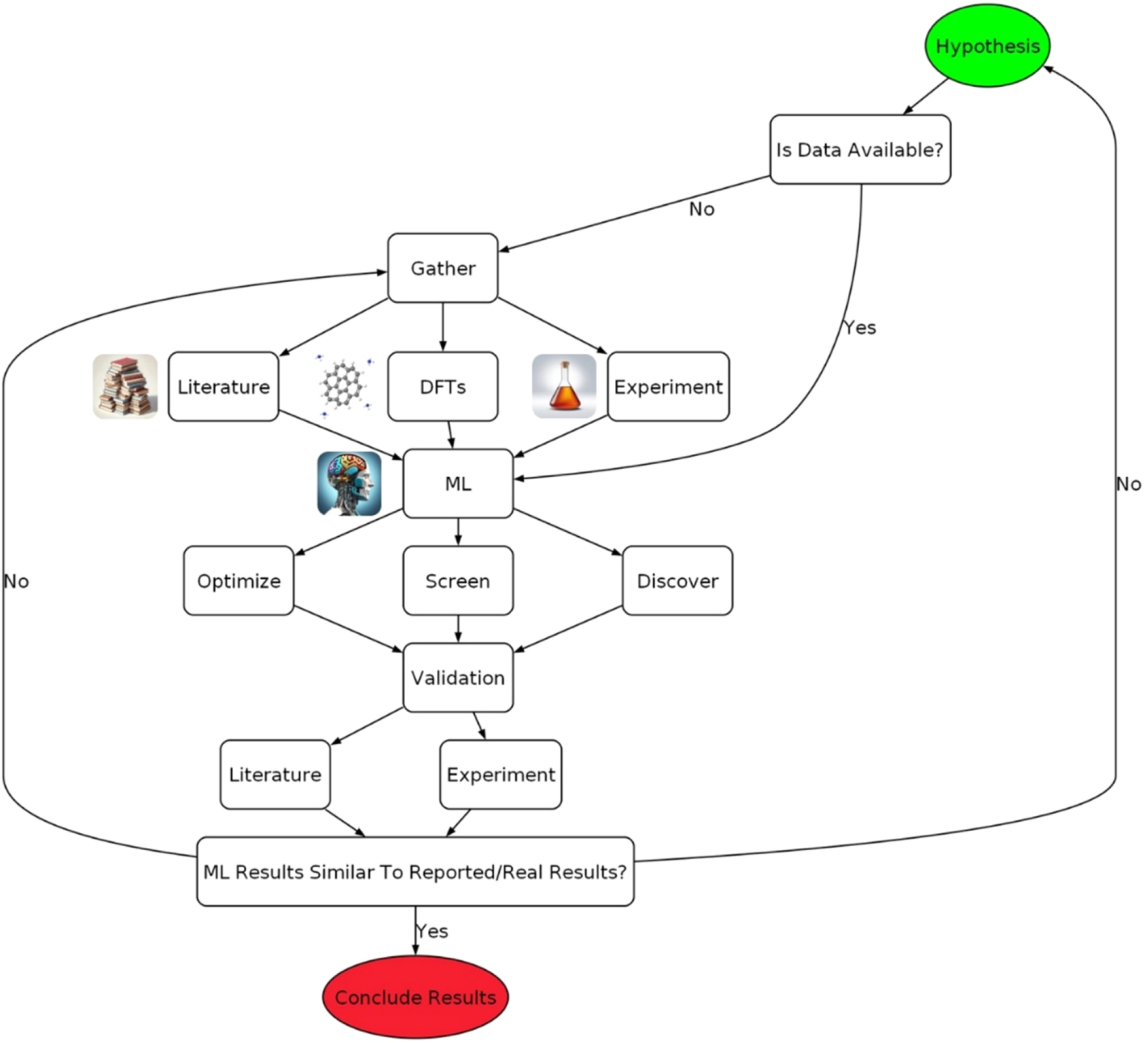
Typical workflow of machine learning incorporated with DFTs, which walks through the beginning of the hypothesis to the conclusion in materials science.

## Machine Learning in 2D Material Energy Applications

Energy conversion is the transformation of one form of energy to another. It is essential for the storage and utilization of excess energy (e.g., solar energy, hydropower, wind energy^120^). The earliest forms of energy conversion were fossil fuels, which were burnt to generate heat to spin turbines for electricity generation.^121^ Here, we observe the conversion of various forms of energy (e.g., chemical, heat, kinetic, and electrical). However, relying on a single source of energy is not sustainable in current times, as there is now a larger demand for energy; coupled with the threat of global warming, an abundant and clean source of energy is needed. This motivates researchers to find new ways to accelerate research in 2D materials for energy applications using machine learning. As discussed, machine learning is a great tool, as it allows the reduction of costs and time for discovery, screening, and optimization. Several applications exist for energy conversion; however, word cloud analysis suggests widely researched topics to be electrochemical catalysts for 2D materials incorporated with machine learning. Diving deeper, electrochemistry refers to the study of electron flow in ionic conductors, which has been applied to many devices such as supercapacitors, batteries, and fuel cells.^122^ Although these devices are similar electrochemically (i.e., they separate ion and electron transport to generate electricity), their mechanisms and storage are vastly different.^123^ Therefore, the use of machine learning can be vastly different, which will be explored in further sections. Photocatalysis, or in this study, photoelectrochemistry, utilizes light and semiconductors to aid in water splitting, which generates H_2_ and O_2_,^124^ which is known as hydrogen evolution. This ties in with the use of fuel cells, which utilize H_2_ and O_2_ for green energy generation.^125^ In the case of catalysts, researchers have given interest in using machine learning, 2D materials, and electrocatalysts to screen materials for the generation of H_2_.^126–128^ Due to the large range of topics, further exploration will be done in their respective sections.

From the gathered literature, a workflow diagram was constructed to aid future researchers in looking to integrate machine learning into electrochemical energy storage research. Figure 10 gives readers a guideline as to how to utilize machine learning for electrode materials, which is the main use of 2D materials in these devices. Electrode materials are first selected to obtain the property; the materials can be existing materials or materials discovered via machine learning. Once the materials are established, the property is then obtained from theoretical calculations (e.g., DFTs), experimentally in laboratories, or predicted from machine learning in a data-driven approach. The properties are then fed into a machine-learning model specifically used to optimize properties. For electrochemical devices, the maximized properties can be capacity, energy density, power density, cycle life, etc. Finally, synthesis can be done from the results or the optimized properties can be used to rediscover electrode materials (i.e., from reverse prediction). This guideline will be utilized as a starting point for further literature review in the section below.

**Figure 10:**
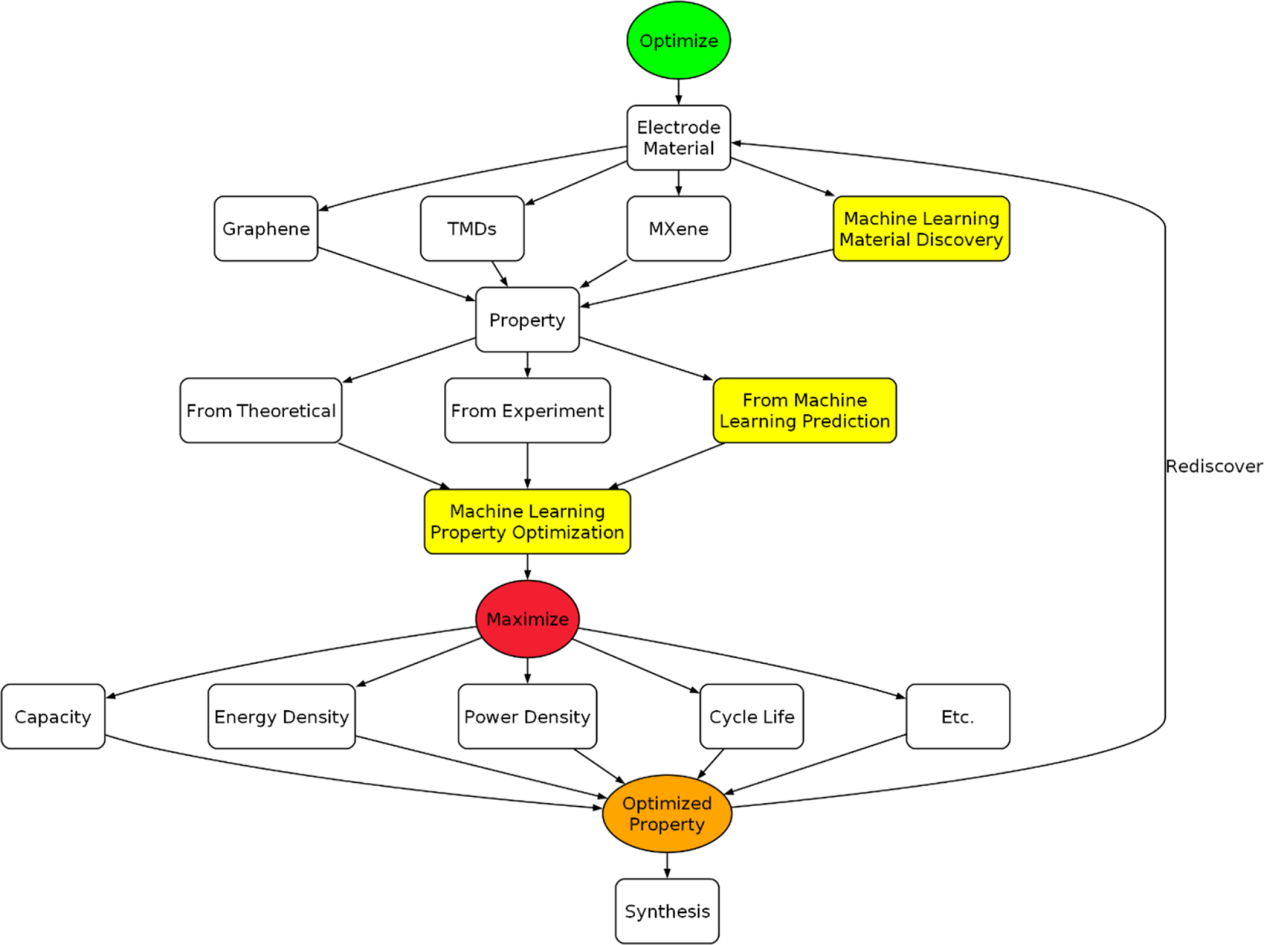
Flowchart of how machine learning is utilized to optimize electrode material.

## Electrochemical Energy Storage

The use of 2D materials in electrochemical energy storage has been studied by various researchers. Notably, the KDD process found its most common usage in fuel cells, batteries, and supercapacitors. Hence, more details will be shown regarding their usage in the electrode of the electrochemical energy storage device. To get a grasp of the details, a summary figure of their usage, benefits, and main focus for machine learning optimization can be found in Figure 11.

**Figure 11:**
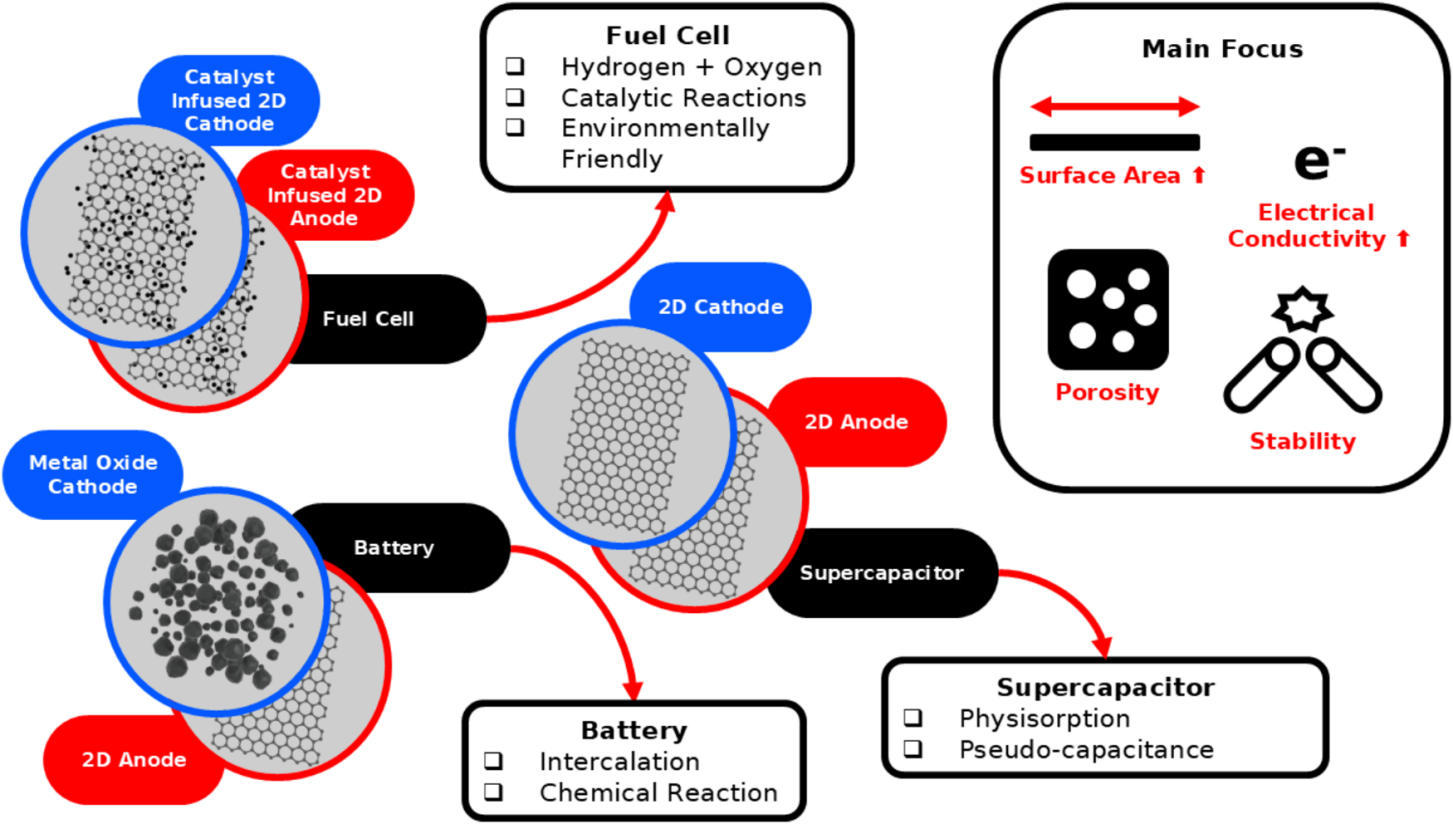
Infographic of the usage of 2D materials in electrochemical energy storage with some notable information such as their mechanism and benefits; their main focus is highlighted to show what machine learning is used to optimize in the specified materials.

Supercapacitor**s** are energy storage devices that store energy through physisorption of ions on electrode surfaces when voltage is applied. This creates positive and negative terminals that attract ions from the electrolyte to the electrode surface. When energy is needed, a load releases these ions, generating electron flow without relying on chemical reactions. Supercapacitors are known for their longevity, cycling over 6.7 million times with minimal capacitance loss,^129^ rapid charging (10 s to 10 min at 2–100 mVs^–1^ ^130^), and the ability to quickly discharge large amounts of energy.

A key limitation of supercapacitors is the surface area of the electrode materials. Carbon materials, including activated carbon, graphene, and carbon fiber, are widely used. To increase performance, doping graphene with heteroatoms like nitrogen, oxygen, or boron enhances its capacitance by adding pseudocapacitance (from chemical reactions) to Helmholtz capacitance (surface area-based), reducing restacking,^131^ and improving wettability.^132^ For example, nitrogen and oxygen doping introduces the pyridine group, enhancing ion adsorption by reducing degradation from surface redox reactions.^133^ However, understanding the effects of surface area, defect ratio, electrolyte concentration, and dopant levels on capacitance is complex and inconclusive.^134^ As a result, data science and machine learning are now key to advancing research into heteroatom-doped supercapacitors. Machine learning is then used to optimize graphene supercapacitor design, particularly the doping conditions. For example, Saad et al. applied machine-learning models such as K-Nearest Neighbors (KNN), Decision Tree (DT), Bayesian regression (BR), and artificial neural networks (ANN) to predict capacitance in heteroatom-doped graphene supercapacitors. Using features like atomic percentages of carbon, nitrogen, and oxygen, surface area, defect ratio, electrode configuration, and pore properties, they found that the ANN model achieved the best fit (*R* ^2^ = 0.88). Their results indicated that nitrogen and oxygen doping had the most substantial effects on capacitance.^135^ Therefore, optimizing conditions (e.g., controlling oxygen content,^136,137^ defect ratio^138^) is essential in obtaining a high-performance supercapacitor. Similarly, Deshsorn et al. used machine learning to optimize heteroatom-doped graphene supercapacitors. They compiled capacitance-related data (e.g., surface area, nitrogen content, defect ratio) and trained a stacking ensemble model to highlight key factors affecting capacitance. They identified sulfur content, graphene surface area, and current density as the top influential features.^13^ This work illustrates how machine learning can guide material optimization for supercapacitors. Furthermore, additional materials other than carbon are being used in supercapacitor space. Here, there is a rise in the number of MXenes and transition metal dichalcogenides being used as the electrode. MXenes are a novel type of 2D material that consists of transition metal carbides, nitrides, or carbonitrides. They consist of M, which is the early transition metals (e.g., Ti, V, Cr, Y, Zr, Nb, Mo, Hf, Ta, and W), X, which is a layer of carbon or nitrogen (C and N), and T, which is the surface terminations (e.g., O, OH, F, Cl).^2^ All of this combines to the formula M_*n* + 1_X*_n_* T_*x*_. Similar to graphene, MXenes have incredible properties for supercapacitor usage (e.g., large surface area, electrical conductivity, tunable surface^139^). However, MXenes are complex in nature to optimize (even more so than graphene) as there is a vast number of parameters present, such as the combination of M, X, T, electrolyte, doping, etc. This then gives rise to utilizing machine learning to aid in the optimization of these materials, whether via the help of DFTs to generate data or traditional experiments. For example, Wang et al. in 2023 used machine learning to aid in the screening of MXene pseudocapacitive abilities for the design of supercapacitor materials.^88^ They utilized DFTs to calculate the MXenes data set, then, applied Sure Independence Screening and Sparsifying Operator (SISSO).^140^ SISSO is a feature generation and selection technique that systematically creates new features by applying mathematical operators (e.g., square root, exponential, and absolute sum) to existing ones. These generated features are then screened and selected by using the Least Absolute Shrinkage and Selection Operator (LASSO), which identifies a sparse set of features that minimize prediction error (e.g., RMSE). By combining feature generation with rigorous selection, SISSO effectively uncovers new interpretable equations to describe complex systems, making it especially valuable for discovering meaningful relationships in high-dimensional data. They discovered that on group-free surfaces (i.e., undoped) both the ion adsorption strength and the density of states 1.0 eV above the Fermi level are crucial in regulating pseudocapacitance. In surface-functionalized cases, electronegativity and specific heat are the key parameters determining the pseudocapacitance. Here, they demonstrate the use of machine learning for both screening of material and the clarification of property significance. Shariq Mohammed et al. in 2024 used machine learning to determine 3 main properties of MXene/graphene nanoplatelets for supercapacitors (specific capacitance, electrical conductivity, and sheet resistance). The data was obtained experimentally via varying the weight percent of graphene nanoplatelets (GNPs) in MXenes. They discovered that artificial neural networks achieved the highest accuracy, with 20 wt % of GNPs in MXene having the highest capacitance prediction of 226.6 Fg^–1^ at 5 mVs^–1^. Furthermore, the model was then used to predict the cyclic stability after 10,000 cycles, which reduces the time of experimentation. This study shows the potential of machine learning in supercapacitor electrode research. Transition metal dichalcogenides or TMDs are 2D materials which is in the form of MX_2_ (M = transition metals and X = chalcogens (S, Se, Te)^3^). They are promising candidates for use as supercapacitor electrode materials as they have high surface area and versatile electronic structure^141^ with an ability for ion intercalation,^142^ which has shown to increase charge storage. This is due to faradaic processes, which enhances the charge storage ability of electrical double-layered capacitance (EDLC).^143^ However, they are not as tunable as MXenes, which offer customizable surface termination T (M_*n* + 1_X*_n_* T_*x*_). TMDs are widely researched in supercapacitor usage; however, to the best of our knowledge, there were no studies involving TMDs, machine learning, and supercapacitors (using the keywords transition AND metal AND dichalcogenide AND supercap* AND machine AND learning). Therefore, it is suggested that the use of machine learning with TMDs is the same with graphene and MXenes, which is the optimization of properties (e.g., capacitance and energy density) and screening of promising materials (e.g., specific usage of MX_2_). From the research done, the development of supercapacitors has greatly benefited from advancements in both material science and machine learning. Traditional carbon-based electrodes, like graphene, offer high surface area and conductivity, but newer materials such as MXenes and TMDs bring unique tunable properties that further enhance energy storage capabilities. In the advancement of supercapacitors, machine learning is playing an increasingly critical role in optimizing these materials by predicting key properties, identifying optimal doping conditions, and discovering new materials with improved performance. As TMDs, MXenes, and graphene are further explored, machine learning will likely continue to streamline the material discovery process, enabling rapid and targeted innovation in supercapacitor technology. This approach not only improves the understanding of how various factors contribute to supercapacitor performance but also shortens the time to develop next-generation energy storage devices. Consequently, integrating machine learning with material science offers a powerful strategy for advancing energy storage solutions to meet the demands of modern applications, from consumer electronics to renewable energy systems.

In addition, **batteries** are electrochemical energy storage devices that generate electricity through chemical reactions. These reactions involve the movement of ions between the anode and cathode of the battery cells^144^ (here, LiMnO_2_ is used as an example). During charging, an applied current prompts the dissociation of the cathode active material (i.e., LiMnO_2_ dissociates into Li^+^, e^–^, and MnO_2_). The resulting lithium ions move internally through the electrolyte to the anode, where they combine with electrons flowing through the external circuit to form lithium–carbon compounds (e.g., LiC_6_ if a carbon material such as graphite is used as the anode). This process is named intercalation of metal ions, which is the main phenomenon for rechargeable batteries.^145^

When the battery is connected to an electrical device, the circuit is complete, allowing the stored energy to power the device by driving electrons through the external circuit. Unlike **supercapacitors**, which store energy via physisorption onto the surface of the electrode without significant chemical changes, batteries rely on chemical reactions where ions and electrons recombine, enabling higher energy density but slower charge/discharge rates (limited by reaction kinetics^146^). However, slower intercalation of lithium ions (e.g., low-temperature operation) into the anode has been shown to cause lithium plating, in which lithium ions accumulate on the surface of the anode instead of penetrating it, reducing the efficiency of ion storage and potentially degrading battery performance.^147^ This lithium plating, in extreme cases, may cause safety concerns, as it can disrupt the separator and cause short circuits. From the mentioned issues, researchers have been trying to discover new 2D materials potentially as the anode or cathode, which can improve charge storage and the desired properties. Therefore, this is a ripe field for machine learning, which can aid in the discovery of new materials. For example, Wan et al. utilized machine learning and DFT calculations to discover new 2D materials, specifically AB_2_ and AB materials, for the anode.^81^ Their goal is to reduce the time and cost of experimentation by first screening promising candidate materials. First, they prescreened materials from potential materials in the 2DMatPedia database (1320 AB_2_ and 593 AB types). Then, they select materials based on specific conditions, such as decomposition energies and exfoliation energies for ease of synthesis from bulk material. After which, 4 criteria were evaluated, which is the capacity (ability to store lithium ions), thermal stability, diffusion barrier of ions, and open circuit voltage. DFT was then used to calculate the capacity, diffusion barrier, and open circuit voltage, while machine learning was used in the thermal stability calculation. This was done to reduce the time of calculation, as large supercells would require tremendous amounts of simulation time. Finally, they screened out TiF, which has outstanding properties such as high electronic conductivity, low energy barrier, high capacity, and high thermal stability. This demonstrates the use of machine learning for battery material discovery. Another study by Sichao Li and Amanda S. Barnard utilized machine learning and MXene DFT data to screen materials that have the desired gravimetric capacity, voltage, and induced charge. They first obtained data containing 360 MXene compounds intercalated with ions from Eames and Islam.^148^ They then encoded the formula M_*n* + 1_X*_n_* T_*x*_Z_k_ (M = Sc, Ti, V, Cr, Zr, Nb, Mo, Hf, Ta; X = C, N; T = H, O, OH, F, NULL; Z = Li, Na, K, Mg; and *n*, *x*, k = 1, 2, or 3) into a vector. Notice that Z is the intercalation element in the MXene. The vector consists of encoded elements present in the formula, for example, [1 0 0 0 0 0 0 0 0 1 0 0 0 0 0 1 1 0 0 0] represents Sc_2_C intercalated with lithium ions. They then used the encoded data to train a random forest machine-learning model. First, they used the features to predict gravimetric capacity, voltage, and induced charge (forward prediction). Then, they trained a model on the prediction target to reobtain the formula M_*n* + 1_X*_n_* T_*x*_ (reverse prediction). This allows both the determination of significant chemical formulas and the discovery of new formulas. They show that not only can machine learning be used to screen data but also it can be used to discover new materials via data. Furthermore, TMDs have been studied in their ability to adsorb and diffuse lithium ions. Chaney et al. did this with machine learning and DFTs to determine if TMDs and Janus TMDs are adequate as an anode material.^149^ Unlike traditional TMDs where the formula is MX_2_, Janus TMDs contain the formula MXY, which results from different chalcogen layers. This provides vertical nonsymmetry and unlocks new properties.^150^ Then, they utilized a linear regression model trained on 72 data points containing TMDs and Janus TMDs information with adsorption energy as their target variable. The model was then used to determine important insight into the adsorption process between lithium ions and TMDs. They found that the inherent dipole of Janus TMDs and the lithium–lithium interaction terms, which vary with concentration, are key factors in determining adsorption chemistry. Finally, suitable candidates for anode materials in batteries are MoSSe and MoSeTe, which provide full coverage of the lithium adsorption on the surface of the material. Their study highlights the use of machine learning in unlocking knowledge relating to the unknown properties of materials. Chiara Panosetti et al. in 2021 studied the process of ion intercalation in graphite structures. Their goal is to enhance the understanding of the underlying mechanism present in lithium-ion batteries such as the complicated fast charging mechanism causing capacity fade and lithium plating. To do this, they utilized density functional tight binding (DFTB), particle swarm optimization (PSO), and Gaussian process regression (GPR). DFTB is a faster approximation of DFTs, which use a precomputed set of parameters. This allows the approximation of electronic interactions instead of recalculation. To calculate the precomputed set of parameters, they utilize PSO parametrization with machine learning repulsive potential obtained with GPR. Combined, this approach saves time and computation of complex systems such as the intercalation of lithium ions inside graphite or, in this case, graphene sheets. This study shows the use of machine learning in understanding the systems of interest with DFTs. Overall, machine learning in 2D material battery research incorporates DFTs to aid in the generation of data. Similar to supercapacitors, they are used to find a suitable anode and cathode for the electrochemical device; however, machine learning in this topic has additional parameters, such as how ions in the electrolyte (e.g., lithium, sodium, magnesium) interact with the electrode, for example, via intercalation and chemical reaction kinetics.

**Fuel cells** are electrochemical devices that use H_2_ and O_2_ in the electrical generation process. This device is different from batteries and supercapacitors in that a catalyst is incorporated into the electrode material, which breaks down the H_2_ stream fed into the system into H^+^ and e^–^. Then, e^–^ travels through an external circuit to supply electricity. The H^+^ then travels through the electrolyte, which combines with O^2–^, which is generated at the cathode with the help of catalysts, to form water. A key advantage of this technology is that it does not generate pollutants, only water and heat. Likewise, the **fuel cell** incorporates 2D materials in the electrode but has additional requirements for high performance. For example, the electrode material must now be a good support for catalysts, meaning that it should not interfere with the catalytic process of H^+^ generation and strongly bind to the catalysts. Therefore, machine learning is then incorporated to find materials with desirable properties for fuel cells. Fu et al. in 2024 used machine learning to discover MXene single-atom catalysts, which can be incorporated into fuel cells.^151^ Single-atom catalysts are isolated single metal atoms dispersed on supports. It achieves high efficiency as the uniform dispersion offers high activity and selectivity.^152^ They screened possible MXene candidates with 7 stable materials being selected, which were Ti_3_C_2_(OH)_*x*_, V_3_C_2_(OH)_*x*_, Zr_3_C_2_(OH)_*x*_, Nb_3_C_2_(OH)_*x*_, Hf_3_C_2_(OH)_*x*_, Ta_3_C_2_(OH)_*x*_, and W_3_C_2_(OH)_*x*_. Then, they identified possible single-atom elements for their cohesion energy and stability. The possible candidates were Zn, Pd, Ag, Cd, Au, and Hg. Machine learning was used to understand the structure–activity relationship. This shows a promising use of machine learning to select and understand electrode material with additional conditions, such as catalyst–support binding. Not only can machine learning be used for material discovery in fuel cells, but it can also be used to investigate fuel cell materials. Vulcu et al. in 2024 used machine learning and Raman spectroscopic technique to predict a nitrogen-doped graphene iron composite catalyst’s state.^153^ Here, they created 3 classes that involve the fresh catalyst, the used catalyst, and the regenerated catalyst. They then trained models with the Raman spectroscopy data representing each class, totaling 33 Raman spectra as the training set and 12 as the test set. The models used were part of the Classification Learner app of Matlab 2018b. This app allows the training and evaluation of many models using Matlab (e.g., support vector, nearest neighbor, and neural networks). They discovered that the best model was KNN using 8-fold cross-validation with an accuracy of 81.8%. Therefore, they successfully tested the use of machine learning with Raman spectra in the evaluation of catalyst states, which resulted in reduced investigation time in various conditions. From the explored literature, it can be seen that machine learning has been prominently used in the optimization and investigation of fuel cells. However, more parameters must be considered as a constraint or feature when using machine learning, such as the binding of a catalyst and support.

## Features in Focus from Machine Learning

To summarize the previous sections, Table 1 is constructed to show which properties can be optimized with machine learning and how they affect each device. Table 1 shows some parameters available for optimization. Additional parameters are also explored, as the literature explored in the previous sections may not cover all properties. For surface area, batteries, supercapacitors, and fuel cells all benefit as the large surface area in the electrode can increase ion intercalation, improve surface charge storage, and increase reaction sites, respectively. Electrical conductivity is another important property, as it allows better electron flow, thus increasing rate capability,^154,155^ improving fast charge and discharge, and increasing electron transport for reactions. Ion intercalation capacity is a unique property that is significant for batteries, which allows higher charge storage and, thus, usage time. However, there have been studies that show intercalation pseudocapacitance, which effectively increases energy density for supercapacitors without losing fast charge capabilities.^156,157^ For fuel cells, ion intercalation may not be necessary, as the main mechanism is based on catalytic activity rather than charge storage. Batteries and supercapacitors essentially do not require any catalytic activity present in their devices, but fuel cells need high catalytic activity for the oxidation of H_2_ and reduction of O_2_ fuels. Porosity for batteries affects the mechanical properties, such as integrity, and the weight, which is affected by how much electrolyte is needed to fill the pores.^158^ Since the electrolyte does not necessarily contribute to charge storage, only acting as an ion transport medium, optimization is key to finding the optimal amount of porosity to not negatively impact applications where weight is a limiting factor (e.g., planes, vehicles). However, supercapacitors rely heavily on surface charge storage, which benefits from porosity. Fuel cells need porosity for gas diffusion, as H_2_ must travel through the electrode to perform a reaction. Chemical stability is needed for all devices to provide a long usage life. Pseudocapacitance is not necessary for batteries and fuel cells but is beneficial in additional charge storage for supercapacitors through reversible redox reactions on the electrode.^159^ For all devices, the optimal level of dopants is crucial as excessive doping can lead to side effects that outweigh its benefits. For example, doping nitrogen on graphene has been shown to increase surface area,^160^ thus, allowing higher surface charge storage with the benefits of some introduced nitrogen groups increasing redox capabilities.^161^ For batteries, doping with K, Al, Cu has been shown to enhance cycle life,^162^ while doping with F in sodium-ion batteries with a Na_0.6_Mn_0.7_Ni_0.3_O_2_ electrode can increase the capacity from the improved electrochemical activity of Mn^3+^/Mn^4+^ and Ni^2+^/Ni^4+^.^163^ Iodine-doped graphene electrode fuel cells have been explored to increase their conductivity; therefore, increasing performance.^164^ Specifically, doping is increasingly explored experimentally, which generates data for use in machine learning doping optimization.

**Table 1:** Properties That can be Optimized for Each Electrochemical Device with Their Respective Effects

**optimize property**	**batteries**	**supercapacitors**	**fuel cells**
**high surface area**	enhances ion intercalation	increases charge storage	increases reaction sites for catalysis
**electrical conductivity**	improves rate capability	allows fast charge/discharge	supports electron transport for reactions
**ion Intercalation Capacity**	energy storage	improves intercalation pseudocapacitance	not required
**catalytic Activity**	not required	not required	essential for reactions (e.g., O_2_ reduction)
**porosity**	mechanical property and weight	benefits charge storage	essential for gas diffusion
**chemical Stability**	stability with electrolyte	stability with electrolyte	resistance to corrosion in harsh environments
**pseudocapacitance**	not required	increases capacitance (optional)	not required
**heteroatom Doping**	enhance capacity	pseudocapacitance redox reactions	conductivity

## Machine Learning for Material Synthesis

Machine learning is transforming the 2D materials field by accelerating discovery and optimizing conditions through predictive insights. However, not only is the application of machine learning to electrochemical devices important but synthesis optimization is also a crucial step for industrial-scale production of materials. Synthesis methods can be categorized into 2 large groups, top-down and bottom-up.^165^ The difference is that top-down approaches create smaller materials from bulk materials in a controlled material removal process, while bottom-up approaches create more complex 2D materials from molecular precursors. Various synthesis methods exist for top-down approaches in 2D materials synthesis, such as mechanical cleavage, liquid-phase exfoliation, and intercalation exfoliation,^166^ while bottom-up approaches include, for example, chemical vapor deposition, physical vapor deposition, wet chemical synthesis,^167^ and flash Joule heating.^168^ The role of machine learning is to aid in the optimization and identification of high-performance variables and methods for efficient material synthesis. Starting off, machine learning has been used to identify promising 3D materials for 2D material synthesis. Vahdat, Agrawal, and Pizzi (2022) utilized machine learning to identify bulk 3D materials, which comprise weakly held layers for 2D material exfoliation.^169^ The descriptors used, or in this case features, are chemical ordering, maximum packing efficiency, and local environmental attributes. Once trained, the model is then used with Shapley Additive Explanations (SHAP), which is a feature-important analysis technique. Inspired by game theory, SHAP assigns each feature a Shapley value, which determines the impact of that feature on a target value (i.e., positive values indicate increase in prediction, negative values indicate decrease in prediction).^170^ Here, they used SHAP to evaluate the features that correlate with the possibility of exfoliation. Their results suggest that maximum packing efficiency, the maximum number of unfilled S states, chemical ordering (first neighbor), mean of the periodic table row, and chemical ordering (third neighbor) are the most important factors in exfoliation. Their study highlights the use of machine learning in carefully selecting materials for synthesis, reducing the time and cost of experimentation. Exploring further, Lu et al. in 2022 used machine learning to control the synthesis of MoS_2_ with chemical vapor deposition.^101^ They used models such as XGBoost, support vector machines, naïve Bayes, and multilayer perception to analyze data obtained from the literature that relate to MoS_2_ synthesis with chemical vapor deposition. Once data was obtained, they labeled data that successfully synthesized MoS_2_ with 1 (can grow) and 0 (cannot grow). The boundary for labeling with 1 and 0 is the size, in which a size less than 1 μm suggests a labeling of 0, and a size more than 1 μm suggests a labeling of 1. The features that were used were the Ar gas flow rate, ramp time, reaction temperature, addition of NaCl, and boat configuration. Finally, they trained the models and observed performance, suggesting that XGBoost performed the best with an area under the receiver operating characteristic curve (AUROC) having a value of 0.91. The ROC curve plots the True Positive Rate (sensitivity) against the False Positive Rate (1 - specificity) at various threshold settings, illustrating the trade-off between correctly identifying positive instances and mistakenly labeling negatives as positives.^171^ A larger area under the curve (AUROC) indicates better model performance, as it shows the model’s ability to effectively distinguish between the classes. Once the best model was obtained (XGBoost), they used the model’s innate feature importance analysis to determine the most important features, which showed that Ar gas flow rate and reaction temperature were the prominent features. To optimize and find the conditions that best succeeds the chemical vapor deposition method, they permuted the features within the input range to obtain 94,26,228 possible combinations, which was then predicted by the model to obtain the highest chance of synthesis. To validate their model, we then used the best conditions to successfully synthesize MoS_2_ experimentally. This study shows that machine learning can reduce an astronomical amount of possible synthesis conditions (in this case, 94,26,228) down to several of the best-performing conditions for synthesis. MXenes synthesis has seen a rise in the usage of machine learning for the investigation of features. To increase the likelihood of successful material synthesis (MXene), Nathan C. Frey et al. in 2019 used positive and unlabeled machine learning, a semisupervised machine learning method that allows the classification of positive data and unlabeled data, which can be positive or negative.^173^ They used this method to learn which samples (structure of MXene and MAX phase) have a high chance of synthesis, in which they labeled positive data as successfully synthesized samples.^173^ Thus, they were able to screen samples, which reduced the time required for trial-and-error experimentation. They started by finding possible candidates from 11 transition M atoms, 12 A group elements, carbon/nitrogen X atoms, and *n* = 1, 2, or 3 layers of X with *n* + 1 layers of M, which yielded 792 single M MAX-phase candidates, after which, the A group was removed yielding 66 single M MXene candidates. Features were then calculated using DFT for the candidates (e.g., bond lengths and formation energy). After this, the model was then trained, and the unlabeled data were segregated. This yielded 111 promising compounds to further screen (e.g., thermodynamics, elastic stability, and phase stability), reducing the candidates down to 18. Their study provides the framework to synthesize the family of 2D MXenes, offering valuable insights and guidance on which material compositions and parameters hold the greatest potential for successful synthesis. Flash joule heating is another method used to mass-synthesize compounds. In particular, this method is useful for the transformation of low-value carbon into high-quality graphene.^174,175^ However, due to the complexities in transforming amorphous carbon into graphene during the heating process, the optimal parameters still remain poorly understood. Luckily, machine learning can be incorporated to discover the best conditions and parameters for synthesis, leading to more efficient and wide-scale use of flash joule heating. Beckham et al. in 2022 followed this principle by utilizing models such as random forest, XGBoost, and linear regression with features such as current density and precursors (e.g., carbon black, pyrolysis ash) to predict the yield of graphene.^176^ Their results suggest that the XGBoost model can learn the inner complexities of the system and can fit the data well (*R* ^2^ of 0.8051). After which, feature importance analysis was performed to obtain the most crucial features, the precursor material, and the stochastic current fluctuation. Their study shows the powerful use of machine learning in analyzing and optimizing 2D material synthesis to obtain high-performing graphene. Another study done on flash joule heating for graphene synthesis was done by Sattari et al. in 2023. Here, machine learning was used to predict the yield of synthesis.^177^ However, they differ in the features used in machine learning and in how they were obtained. The authors utilized direct processing variables and indirect as the features, with physics-informed variables being used to predict the graphene yield. The direct variables contain information about the material (e.g., material type, surface area, and mass) and process (e.g., voltage and reaction atmosphere). The indirect variables consist of features derived from current (such as final current, peak current, and charge density) obtained through proxy machine-learning models, as well as reaction temperatures simulated via multiphysics modeling. This allows more information to be made available to the machine-learning model, increasing the *R* ^2^ to 0.81 (with indirect variables) for the model utilized. This study shows the usefulness of machine learning in both learning the prediction environment and also generating features (in this case, the current). Looking at chemical exfoliation (i.e., liquid-phase exfoliation), Ton et al. in 2020 used machine learning to understand the underlying synergistic effects of the solvent and solvent mixtures on the exfoliation of graphite.^178^ Here, they identified synergistic effects between functional groups (e.g., aromatic, amine). Additionally, the mixing of solvents provides more benefits than that of single solvents. The screening results were then applied in a new machine learning approach based on the Dempster-Shafer theory to systematically analyze synergistic effects and suggest novel mixtures. Their study shows that the integration of experimental and data-driven methods proved to be highly effective for identifying synergistic solvent mixtures. Machine learning is establishing a solid foundation in the field of material synthesis, enabling optimization of synthesis conditions that reduce time and advance 2D material production to an industrial scale. This approach also lowers research costs, freeing resources for larger projects within the field. Overall, machine learning is being applied across various synthesis methods, from top-down to bottom-up approaches, offering valuable insights through data-driven analysis.

## Model Feature Importance Analysis

To extract important parameters of machine learning, feature importance analysis is vital in a researcher’s toolkit. Molnar stated in the book “Interpretable Machine Learning (2020)” that machine learning needs to be understood for humans to identify the problem and solve it.^179^ It is therefore important for machine learning to be combined with human knowledge (i.e., expert domain knowledge) to confirm and validate the model results, leading to desired models not only having high predictive power but also high interpretability. However, complex problems require complex solutions. In this case, the complexity of 2D materials requires an abundance of data and features (parameters). Consequently, complex models are often used to achieve high prediction accuracy, although this can come at the expense of model interpretability. Luckily, Christoph Molnar stated that there are 2 major ways to increase interpretability: (1) using interpretable models and (2) using interpretability techniques (feature importance analysis). Interpretable models, such as linear regression and decision trees, are excellent interpretable models. Linear regression shows weights that can be analyzed for positive or negative contribution,^180^ and decision trees contain split paths^179^ that can be analyzed to understand the model’s decision process. However, they may suffer from overfitting^181^ if not tuned properly *via* the usage of regularization^182^ or pruning.^183^ Therefore, this section will focus more on interpretability techniques, as they can be model agnostic, meaning they can be used in any type of model. This brings the advantage of being able to use these techniques on complex models, thus tackling a wider range of problems.

The first technique to be explored is the **Shapley Additive Explanations** (SHAP). SHAP was developed to make complex model predictions more understandable by assigning Shapley values to features, indicating each feature’s contribution to the prediction. A positive Shapley value shows that a feature positively influences the target variable, while a negative value suggests a detrimental effect. For an intuitive idea of the process behind SHAP, a table with pseudocode can be seen in Table 2, showing a simplified process behind SHAP. First, a feature is selected, then all marginal contributions of the feature are computed and averaged, giving a Shapley value.

**Table 2:**
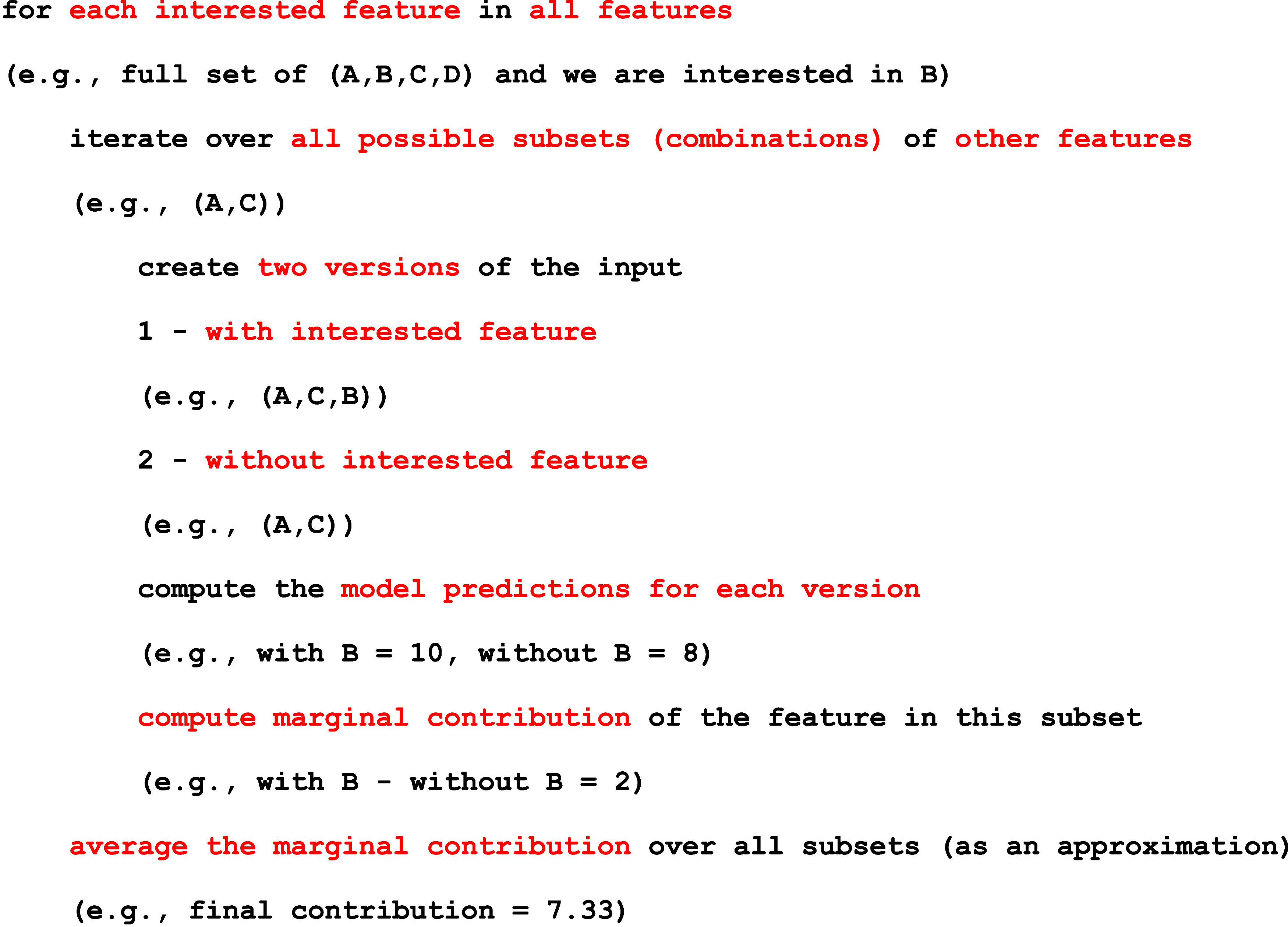
Pseudocode Example of the Process and Intuition behind Computing SHAP

An example of a real applied workflow can be observed in Figure 12, where a background data set and the test data to a normal data-trained model in conjunction with the SHAP process. After this, positive or negative Shapley values can be obtained to plot the trends. An example usage is in Figure 13, which shows the Shapley values of surface area obtained from a stacking model in Deshsorn et al.’s study.^13^ In this analysis, the user can observe the trends of the Shapley values (e.g., the higher the Shapley value, the larger the surface area) while accounting for the interaction of other variables processed by the machine-learning model. This essentially reduces complex, multidimensional features to a level that humans can understand and interpret the results. To improve the accuracy of Shapley value approximations while reducing computational demands, Lundberg and Lee (2017) introduced KernelSHAP, which employs a specialized weighted linear regression for efficient Shapley value estimation.^170^ To demonstrate the usage of SHAP, Manna and Pathak (2024) utilized machine learning and SHAP to identify suitable cations for graphite intercalation in dual ion batteries.^184^ Using their best model, XGBoost, they predicted the binding energies of various cations during intercalation. After which, SHAP was used to highlight the most important features that contributed to the prediction, which were the number of cations, the stage of intercalation, and the lowest unoccupied molecular orbital energy (LUMO). Additionally, explanations as to which factors affect binding energies can be used to optimize and discover novel cations used in dual ion batteries by observing the Shapley values that were obtained. The Shapley value clusters show that high numbers of cations are not beneficial to binding energy (roughly −0.5 Shapley value). However, a low number of cations achieves a high contribution to binding energy (highest positive Shapley value of roughly 2.5). To increase the trustworthiness of results, their expert domain knowledge is then used to explain this phenomenon. This demonstrates the use of SHAP not only to discover high-impact features but also to understand whether high or low values of a variable positively or negatively affect the target variable, in this case, binding energy.

**Figure 12:**
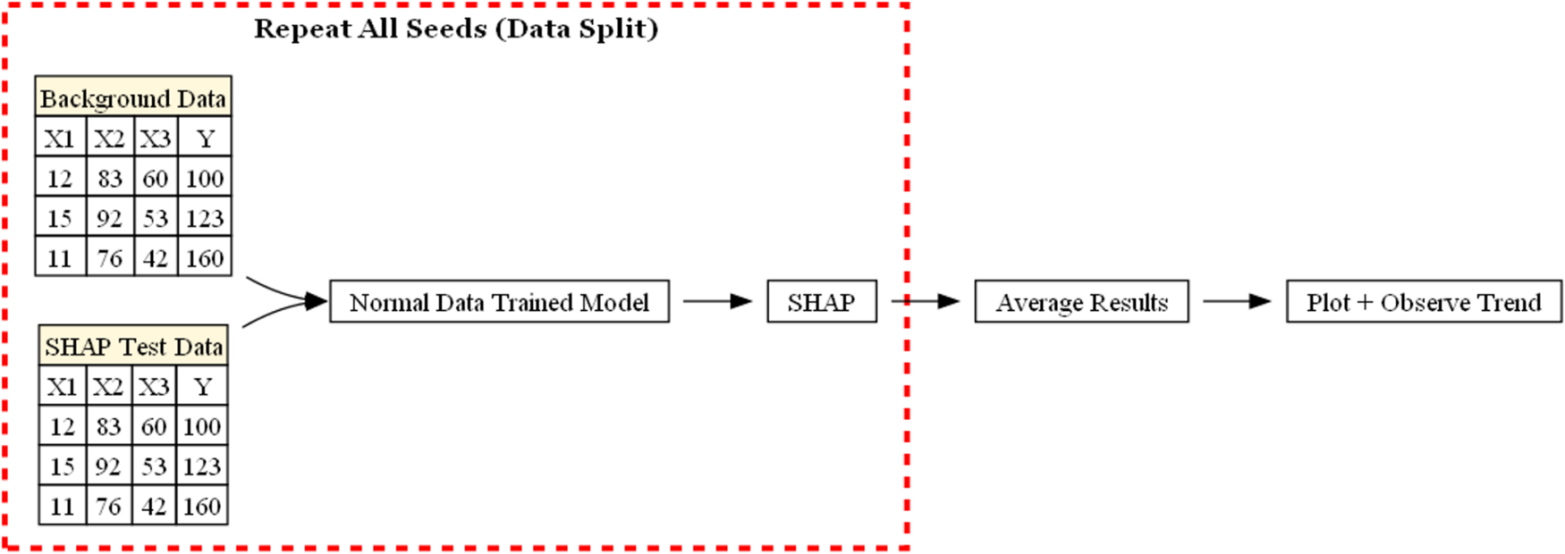
Demonstration of the workflow of SHAP, which involves feeding a background data set and the test data to a normal data-trained model in conjunction with the SHAP process, which is then plotted and observed.

**Figure 13:**
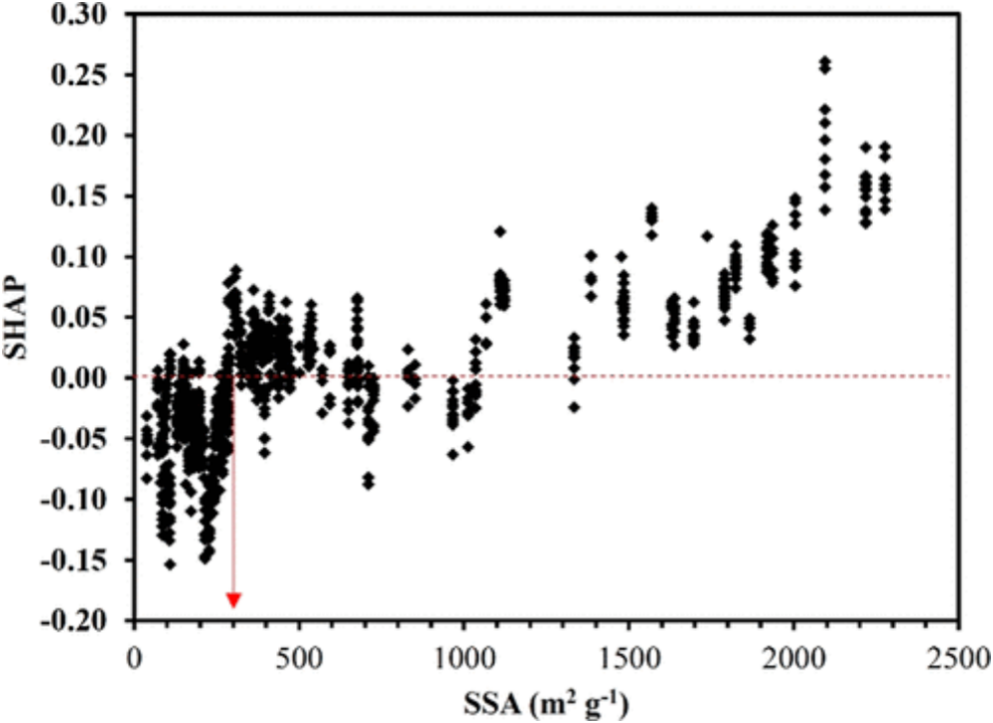
Shapley value (*y*-axis) compared to the surface area (*x*-axis) of a stacking model trained on graphene supercapacitor data. Reproduced from^13^. Copyright [2025] American Chemical Society.^13^

Another interesting model-agnostic technique is the **Partial Dependence Plots** (PDP). PDP analyze feature importance by keeping all input variables constant except for the feature of interest, which is varied.^185^ This technique allows researchers to observe trends in the predictions as the selected feature changes, helping to identify its optimal value. The values for the feature of interest typically span from the zeroth to 100th percentiles at 10% intervals, while other features are held at a certain value (e.g., mean, median). For readers, an intuitive idea of the process behind PDP can be seen in Table 3. First, a feature is selected, then, a new data set is created with the percentile values of the feature. After this, the values of all other features are fixed at a certain value (e.g., mean). Finally, each row of the new data set is predicted using a trained model, which yields a trend of the feature values from the zeroth percentile to the 100th percentile.

**Table 3:**
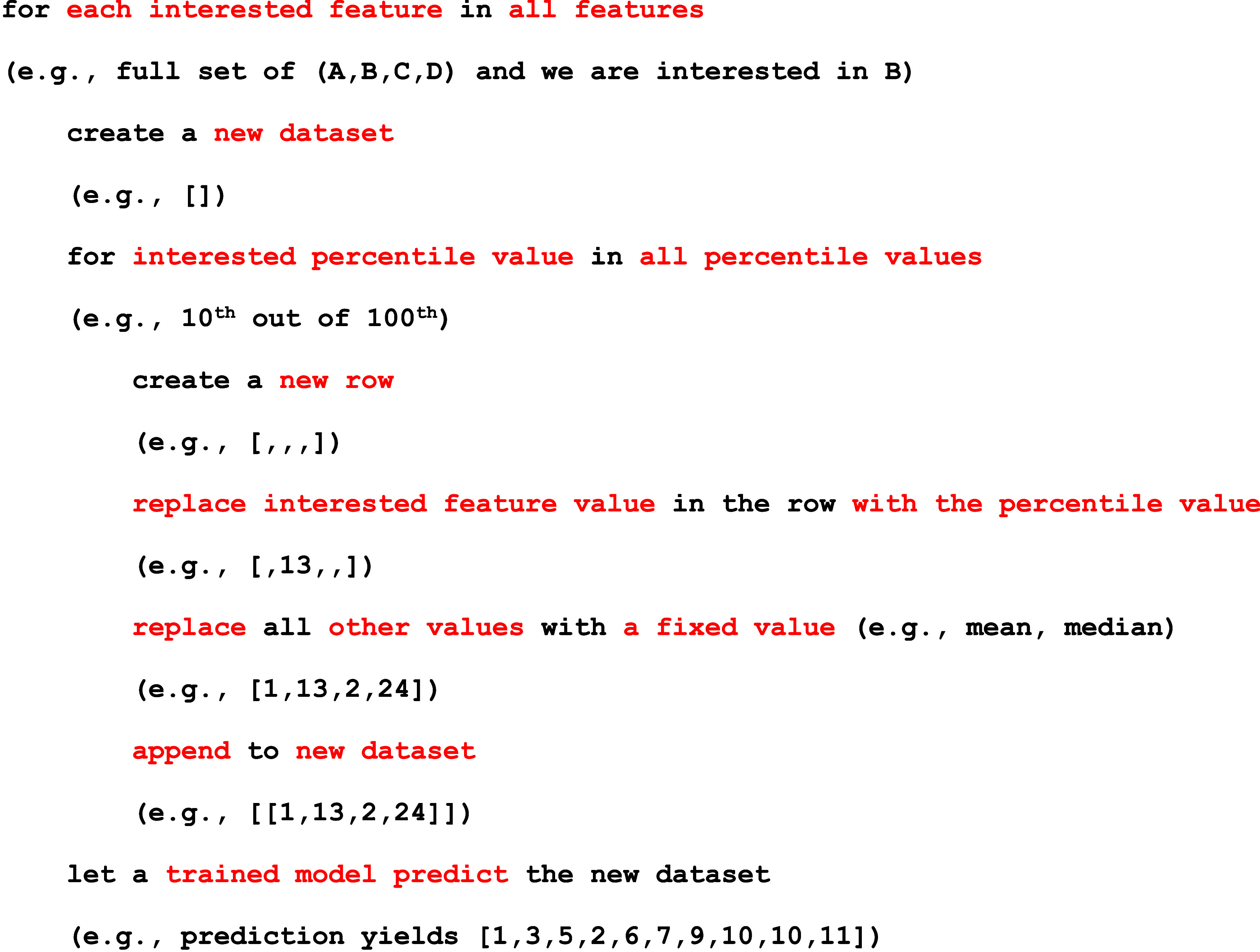
Pseudocode Example of the Process and Intuition Behind Computing PDP

Figure 14 shows an example of the process in a publication, where a single feature is set to a value in a 0 to 100 percentile of that feature range while keeping other features at a constant (e.g., the mean). The new data set is then predicted via a normal data-trained model to observe the trends. Deshsorn et al. in 2024 studied the effects of machine learning noise and its effect on prediction and feature importance analysis with heteroatom-doped graphene supercapacitors.^14^ They tested various techniques, such as contour plots, SHAP, PDP, and how the prediction changes based on increasing noise. Specifically, PDP was tested by fixing noninterested variable at its scaled value of 0.5, while the interested variable was varied in the scaled range of 0 to 1 with a 0.1 value interval. Observing their trends at 0% noise (in Figure 15), current density showed an exponential decay curve similar to other studies done with supercapacitors.^186,187^ Additionally, as noise increases, the trend starts to decay and is more random in nature. Therefore, PDP can be used to observe trends of machine-learning models while accounting for some effects of other features, which can be difficult without specific techniques.

**Figure 14:**
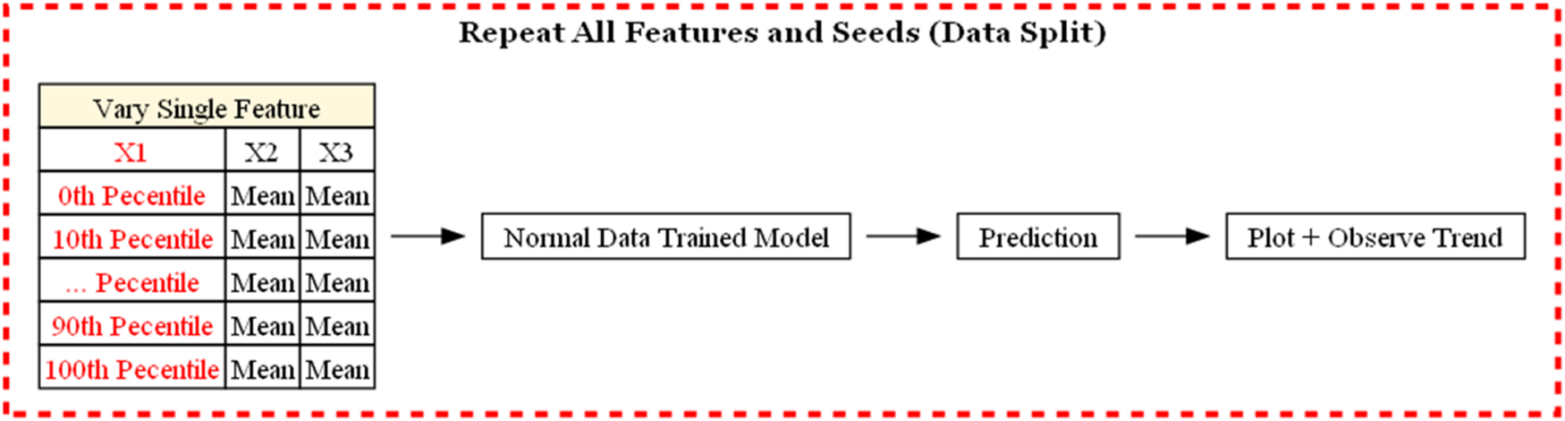
Demonstration of the workflow of PDP, which involves taking a normal data set and varying a feature in a percentile range (0 to 100) while keeping other features constant, after which, the shuffled data are predicted with a normal data-trained model to obtain the prediction.

**Figure 15:**
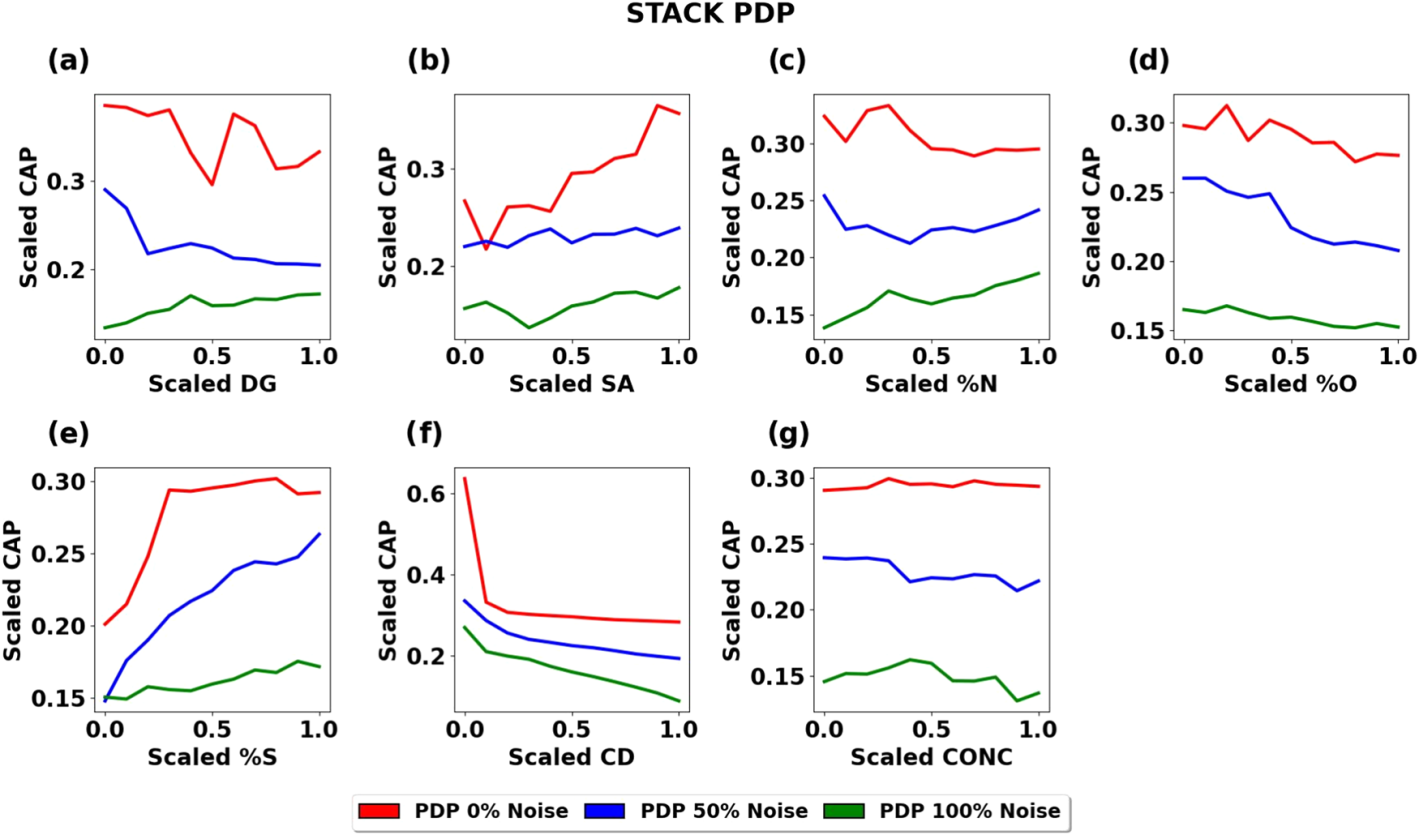
PDP of a stacking model and the effect of noise in the data set for (a) defect ratio, (b) surface area, (c) % doped nitrogen, (d) % doped oxygen, (e) % doped sulfur, (f) current density, and (g) electrolyte concentration. Reproduced with permission from^14^. Copyright [2025] [Elsevier].^14^

The last technique that will be discussed is **Feature Permutation Importance** (FPI). FPI assesses feature importance by randomly shuffling values within input features and measuring the effect on model predictions.^188^ After shuffling, the model is retrained on the permuted data set, and its predictions are compared to those from the original, unaltered data using error metrics like mean absolute error. A larger increase in error from shuffling indicates a more influential feature, while a smaller error change suggests that the feature has a weaker relationship with the target variable (e.g., capacitance or energy density), as shuffling has little effect on the predictions. A simplified process can be seen in the pseudocode in Table 4. First, the interested feature column is shuffled, after which, a trained model predicts the shuffled data set and the performance metric is obtained. Lastly, it is then compared to the performance metric of a normal, nonshuffled data set.

**Table 4:**
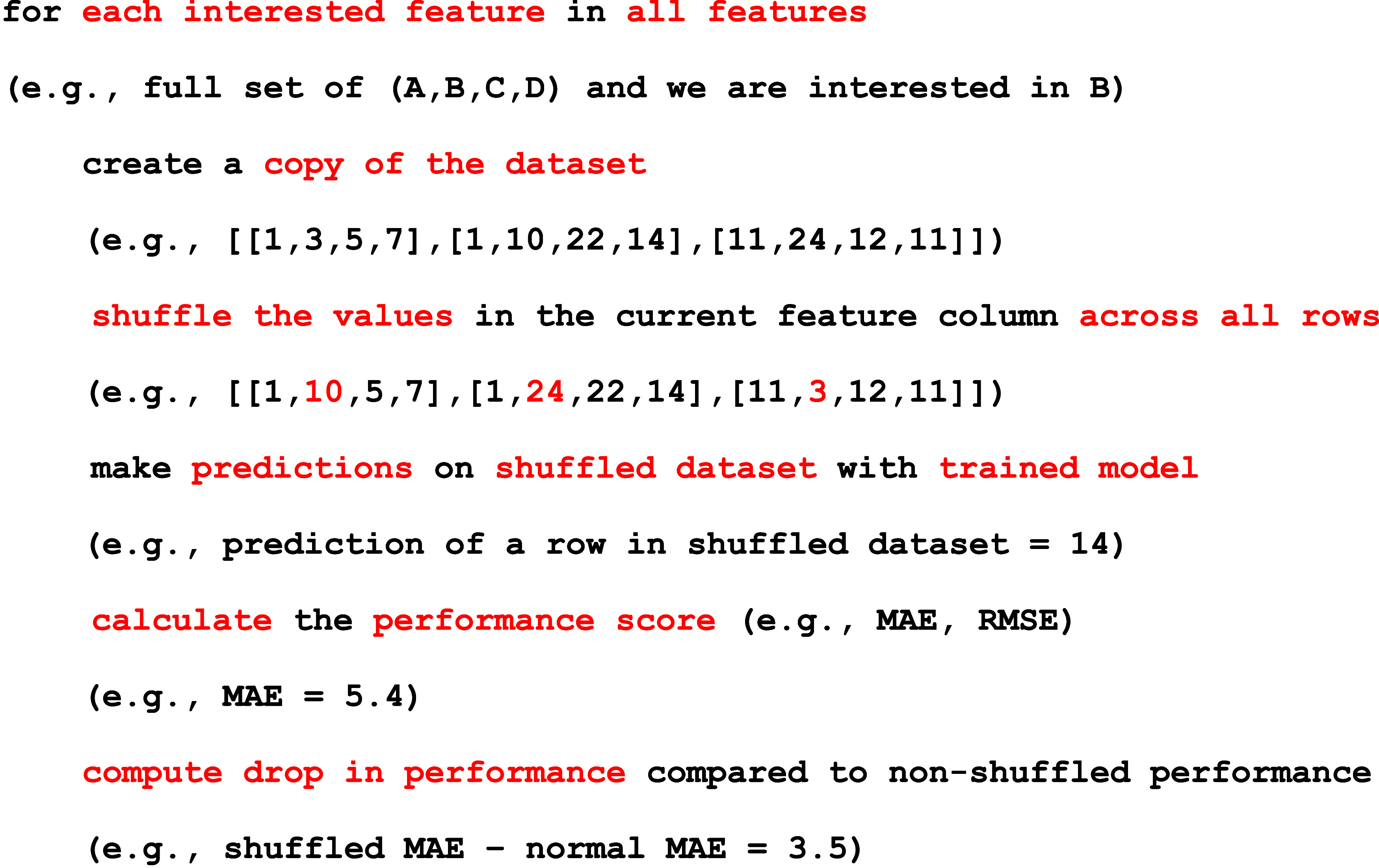
Pseudocode Example of the Process and Intuition Behind Computing FPI

An example of a real applied workflow can be seen in Figure 16, where a feature of a normal data set is shuffled, after which, the shuffled data is predicted with a normal data-trained model to obtain the prediction compared with the real results and normal data prediction. Orts Mercadillo et al. in 2023 utilized FPI to study important Raman spectra features that affect the model’s ability to classify baseline and fluorinated graphene nanoplatelets materials.^189^ Here, they utilized random forests to perform features with a high permutation importance. From their results, it was observed that the initial properties of the material influence which Raman features show the most change after functionalization.

**Figure 16:**
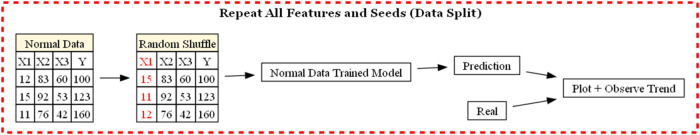
Demonstration of the workflow of FPI, which involves taking a normal data set and shuffling a feature, after which the shuffled data are predicted with a normal data-trained model to obtain the prediction compared with the real results and normal data prediction.

Feature importance analysis techniques like SHAP, FPI, and PDP offer significant advantages for studying 2D materials by revealing how individual features or properties influence a model’s predictions. Optionally, other feature importance analysis tools that are useful for researchers that are not covered are (1) Genetic Algorithms,^190^ which can find the most important feature by identifying the most frequent feature in high target values, top performing subsets, (2) Local Interpretable Model-agnostic Explanations (LIME), which perturbs input data locally and fits a simple model (like linear regression) to explain predictions around a specific point,^191^ and (3) Drop-Column Importance (DCI), which compares the drop in performance after removing a feature when training a model.^192^

Overall, in the context of 2D materials, where properties (e.g., surface area and defect ratio) can dramatically affect performance, these techniques provide insight into which factors most strongly impact target outcomes such as conductivity, capacitance, or energy density. This understanding helps prioritize key features, guide experimental efforts, and optimize material properties with greater accuracy, ultimately accelerating the development of 2D materials for applications in energy storage, catalysis, and electronics.

## Artificial Intelligence (ChatGPT) Versus Human Outlook for Future Trend of 2D Materials in Electrochemical Applications

In addition to manual data mining processes such as KDD, the authors are interested in the perspective of artificial intelligence (**OpenAI GPT-4 model**) and its outlook for the future trend of 2D materials in electrochemical applications. Therefore, the authors asked the OpenAI GPT-4 model a question in a new chat window, ***“With the rise of artificial intelligence and large language models being used for research, what can you tell us about the future trends of using machine learning in 2D materials in electrochemical applications research? Limit the response to 300–500 words.”***. After which, the authors utilize their domain knowledge, insights, and data mining information to criticize the model’s output. This brings unique perspectives from artificial intelligence and can increase the model’s reliability and trustworthiness if its analysis corresponds with the findings in this research. All in all, this allows the general public and researchers alike to comprehend how far and advanced large language models have become. Here, the AI provided 5 sections of responses, which will be analyzed accordingly. Following the response of the AI, a table to summarize the results of their perspective is shown in Table 5.

**Table 5:** Highlights of Asking the AI Regarding the Topic ***“With the Rise of Artificial Intelligence and Large Language Models Being Used for Research, What can you Tell us About the Future Trends of Using Machine Learning in 2D Materials in Electrochemical Applications Research? Limit the Response to 300–500 Words.”*** Which is Separated into 5 Sections Given by the AI

**AI-generated sections**	**AI response highlights**
**materials discovery and design**	traditional methods are slow and cost-intensive
	ML quickly screens materials
	predict properties (e.g., conductivity, stability)
	accelerate development of fuel cells, batteries, and supercapacitors
**high-throughput screening and optimization**	combining ML with high-throughput computational tools
	faster than traditional experimental methods
**improved predictive models for electrochemical performance**	reinforcement learning can adjust parameters based on feedback from experiments to continuously improve predictive accuracy
	reveals complex phenomena such as ion transport, charge distribution, and degradation
**enhancing catalysis and energy storage**	TMDs, graphene, and their hybrids, are candidates for enhancing catalytic activity and energy storage
	ML improves the design of electrodes in batteries or supercapacitors by predicting ideal parameters
**data-driven research pipelines**	experimental data, computational predictions, and ML models are seamlessly integrated
	rapid translation of findings from the laboratory to commercial applications

In the **first section**, the AI describes the use of machine learning materials discovery and design. It describes the use of traditional methods (i.e., traditional statistical analysis) as being too time-consuming and costly. This aligns with the findings in this study, where most literature states that they utilized machine learning to reduce the time of research (e.g., through screening) and cost reduction (e.g., through optimization). In addition to this, it mentions the use of models to explore large databases, which is also mentioned in this study. Surprisingly, the model mentions batteries, supercapacitors, and fuel cells as the main examples of electrochemical usage (in accordance with data mining). The model also mentions the optimization of conductivity, stability, and catalytic activity, which is discussed in the Features in Focus from Machine Learning section. In the **second section**, the AI highlights the use of machine learning in screening a large quantity of materials and experimental results to identify usable 2D materials. It mentions combining high-throughput computational tools, likely referring to density functional theory (DFTs), with machine learning to evaluate materials under different conditions, which is similar to exploring the synergistic interaction of parameters (e.g., varying both temperature and pressure at the same time). This is also mentioned in the literature reviews in previous sections, especially the usage of DFTs with machine learning. In **third section**, the AI mentions using **reinforcement learning**, a technique that includes letting a model learn using a reward-based system. Reinforcement learning has a wide range of usages (e.g., in robotics,^193^ and industrial process controls^194^); however, using the model to continually adjust parameters based on feedback from experiments is a relatively novel usage of reinforcement learning in the electrochemical field. This unique approach can be utilized to improve energy storage devices, for example, by balancing the charge/discharge rate or by dynamically adjusting the current density for supercapacitors. In the **fourth section**, TMDs and graphene are highlighted as potential candidates to use as catalysts and in energy storage. In energy storage, this is partly due to their ease of functionalization (i.e., doping), which has been proven to improve the charge storage of supercapacitors through Faradaic reactions. Here, machine learning is described as a tool to optimize the synthesis of these materials, tailoring their properties for specific usage, for example, in the graphene doping optimization using machine learning section, which is in accordance with this study’s findings. Lastly, in **fifth section**, the AI highlights the integration of machine learning, experimental, and computational data in a smooth, data-driven research pipeline. It highlights that this approach will promote a more cooperative, streamlined, and transparent research environment, facilitating the swift transition of discoveries from the lab to real-world applications. This is true in certain studies found in this review, where the full cycle of data acquisition, machine learning, and synthesis is all reported in a single study (e.g., Pradhan et al.^116^).

Overall, AI has shown great ability to describe the trends of certain topics. However, as of now, AI does not delve deep into the literature, unlike humans. This in turn, demonstrates that researchers cannot rely solely on a model; rather, domain knowledge must be incorporated into the model’s analysis. Nevertheless, the cooperation of researchers “fact-checking” the AI and AI introducing novel ideas for researchers (e.g., reinforcement learning) can prove to be a valuable tool for researchers who are often “stuck” on a topic, aiding in the overall advancement of the field.

## Limitations and Future of this Study

Although this study dives into the use of data mining to advance research, a discussion of the limitations of data bias and incomplete data sets must be done. First, data bias may be present as certain stopwords can have an impact on the analysis. For example, some elements reported in articles are reported in their shortened form (e.g., oxygen as O); therefore, stopwords or conditions that remove “O” from the data set can lead to bias in the analysis (e.g., nitrogen is found more than oxygen). Another discussion is of incomplete data sets. This will prove to be a problem as scientific progress is advancing at a remarkable rate. This leads to new literature always being found and added to databases. Therefore, a data mining review will never be fully complete. Expectantly, future researchers can improve on the methodologies in this review, solving the data bias problem (e.g., better filtering) and incomplete data set problem (e.g., daily updating plots on a Web site).

Another discussion is the usage of machine learning and the current limitations of machine learning. Even though data are sufficient in the present, for hypothesis-driven research, data can be difficult to obtain. For example, doing research on a new and novel topic will leave the researcher with no previous data on which to train the model on. This brings up the importance of combining both traditional lab-based experiments with computational calculations and machine learning, as suggested by the AI, which allows data to be seamlessly integrated into computation and machine learning. However, much better equipment for data acquisition (e.g., better sensors and better lab setup for flawless data pipeline) is of concern. Moving forward, laboratories and experimental sites may have to concern themselves with how to store and move data along with how to perform experiments. Additionally, laboratories and experimental sites may need to have researchers both skilled in experimental work and computational work, motivating researchers to be multidisciplined and inspiring a change in the educational sector to incorporate these skills.

Even though these limitations exist, future researchers can use these data mining reviews as a time capsule, revealing how much research and the challenges in the field have changed in the past compared to the present.

## Conclusions and Outlook

Machine learning is a useful tool that can be incorporated into 2D materials design. It is especially useful in the optimization, discovery, and screening of materials. In this study, a historical deep dive into the use of machine learning in 2D materials for electrochemical energy applications is explored. The Knowledge Discovery in Databases (KDD) method was used to reliably guide this study, beginning with an understanding of the domain to the utilization of knowledge. The data used in this study was obtained from Scopus, a database for literature, which can be queried with various keywords to search for publications. The keywords 2d OR ″two dimen*″ OR ″2 dimen*″ AND material* AND “machine learn*” AND elec* were used, which yielded 365 data entries with information such as the abstract, corresponding author address, keywords, and year of publication. Data mining methods such as word clouds, co-occurrence networks, and heat maps analysis were utilized to obtain information such as the highest cited publication, the country with the most citations, the earliest publication, and so on, which revealed insights into how machine learning usage has progressed in recent years. Overall, a large number of studies utilize **DFTs** with machine learning; therefore, these fields will go hand-in-hand with the future of material science. Not only is machine learning being used with computer-calculated data, it is also being used with experimental data; however, obtaining a large data set is no easy task. Accordingly, researchers must scrape data points available in various publications, which can take a significant amount of time; however, it requires less time than performing all experimentations from scratch. In turn, research done in the past is not only a vital source of knowledge, but it is now also an invaluable source of data for machine learning. Afterward, the key insights obtained were used to review additional papers related to the topic, with the author guiding researchers on how machine learning is utilized. The topics discussed were **density functional theory**, **energy conversion**, **supercapacitors**, **batteries**, **fuel cells**, **parameter optimization**, **synthesis**, and **feature importance techniques**. From these topics, parameters that can be optimized were summarized into a table, with a complementary figure to guide researchers in incorporating machine learning in electrode optimization.

Overall, this study not only determined that machine learning’s insight into the future trends of machine learning usage in 2D materials for electrochemical applications are in accordance with the literature gathered but also has the capacity to rapidly screen and evaluate countless material candidates that will unlock materials with optimized surface area, conductivity, and mechanical strength, accelerating innovation at a previously unthinkable pace. This technology reduces experimental costs and time while leveraging past research as a rich data source, redefining how we obtain collective knowledge. As machine learning models grow increasingly precise and interpretable, this study aspires to be a valuable resource, guiding both experienced or beginning researchers toward a more efficient, sustainable, and electrically advanced future through the power of data-driven 2D material design.

## Supplementary Material



## Data Availability

The code and data are available on the 1st author’s GitHub (https://github.com/Demodesu). For the specific repository, please visit https://github.com/Demodesu/Data-Mining-Into-Machine-Learning-Aided-2D-Material-Research-In-Electrochemical-Application.

## Notes

Scopus database showing the literature information e.g., author, title, year, keywords including abstract, and DOI. The following files are available free of charge (XLSX)
